# Breaking Bad: How Viruses Subvert the Cell Cycle

**DOI:** 10.3389/fcimb.2018.00396

**Published:** 2018-11-19

**Authors:** Ying Fan, Sumana Sanyal, Roberto Bruzzone

**Affiliations:** ^1^HKU-Pasteur Research Pole, LKS Faculty of Medicine, School of Public Health, The University of Hong Kong, Hong Kong, Hong Kong; ^2^MRC Protein Phosphorylation and Ubiquitylation Unit, School of Life Sciences, University of Dundee, Dundee, United Kingdom; ^3^LKS Faculty of Medicine, School of Biomedical Sciences, The University of Hong Kong, Hong Kong, Hong Kong; ^4^Department of Cell Biology and Infection, Institut Pasteur, Paris, France

**Keywords:** viruses, cell cycle, checkpoint, infection, life cycle, host-pathogen interactions, phosphorylation, degradation

## Abstract

Interactions between the host and viruses during the course of their co-evolution have not only shaped cellular function and the immune system, but also the counter measures employed by viruses. Relatively small genomes and high replication rates allow viruses to accumulate mutations and continuously present the host with new challenges. It is therefore, no surprise that they either escape detection or modulate host physiology, often by redirecting normal cellular pathways to their own advantage. Viruses utilize a diverse array of strategies and molecular targets to subvert host cellular processes, while evading detection. These include cell-cycle regulation, major histocompatibility complex-restricted antigen presentation, intracellular protein transport, apoptosis, cytokine-mediated signaling, and humoral immune responses. Moreover, viruses routinely manipulate the host cell cycle to create a favorable environment for replication, largely by deregulating cell cycle checkpoints. This review focuses on our current understanding of the molecular aspects of cell cycle regulation that are often targeted by viruses. Further study of their interactions should provide fundamental insights into cell cycle regulation and improve our ability to exploit these viruses.

## Introduction

The study of host-pathogen interaction and the search for host factors that are crucial for virus life cycle is critical to the understanding of viral pathogenesis, and may shed light on the development of therapeutic approaches. Viruses are obligate intracellular parasites that constantly evolve strategies to subvert their host cellular environment. As survival of viruses depend on the ability to replicate in living organisms, it is not surprising that they are able to arrest or promote cell cycle progression, depending on the purpose, to their advantage. Viruses that are associated with malignancies, such as human T-cell leukemia virus type I (HTLV-1), Epstein-Barr virus (EBV), human papilloma virus (HPV), and Kaposi's sarcoma-associated herpesvirus (KSHV), have long been known to antagonize cell cycle checkpoints for neoplastic transformation of infected cells (Dyson et al., 1989; Moore and Chang, 1998; Knight and Robertson, 2004; Iwanaga et al., 2008). Thus, Simian virus 40 (SV40) and HPV promote entry of host cell cycle into the S phase as viral DNA replication depends on the host DNA replication machinery (Lehman et al., 2000; Banerjee et al., 2011). In contrast, human immunodeficiency virus type 1 (HIV-1) infection of T lymphocytes results in a cell cycle arrest in G2, suggesting that HIV-1 may disrupt T-cell-mediated immune response by preventing its clonal expansion (Zimmerman et al., 2006). The avian coronavirus infectious bronchitis virus (IBV) also promotes favorable conditions for viral protein synthesis and, hence, progeny virus production, by inducing cell cycle G2/M phase arrest of virus-infected cells (Dove et al., 2006).

The investigation of mechanisms viruses employ to hijack host cell cycle is, therefore, of significance not only for insights into the intracellular viral lifecycle, but also for development of strategies to counteract viral infection. Viruses associated with human cancer have been the focus of scores of studies that have led to the identification of cellular networks regulating the cell cycle, including the discovery of oncogenes and tumor suppressor genes (Bishop, 1991; Weinberg, 1991). While discussion of their manipulation of the cell cycle is inevitable, oncogenic viruses will not be the focus of this article and the reader is referred to a number of excellent reviews on this topic (Weinberg, 1997; zur Hausen, 2001; Chang et al., 2017). Rather, we summarize and discuss here current concepts of the mechanistic features that non-oncogenic viruses adopt to modify host cell cycle machinery.

## Overview of the cell cycle

Cell cycle, or cell-division cycle, is a highly regulated process during which a cell undergoes duplication and division leading to the generation of two daughter cells. The eukaryotic cell cycle is generally divided into four stages: gap 1 phase (G1), synthesis phase (S), gap 2 phase (G2), and mitotic phase (M). In between M and G1, a cell may enter a quiescent state called gap 0 phase (G0), during which cells are neither dividing nor preparing to divide.

G1 is the phase during which metabolic changes take place to prepare the cell for division. Events that take place in G1 include growth in cell size as well as mRNA and protein synthesis. Initiation of G1 phase requires growth factor stimulation and a continuous supply of growth factor until the cell cycle passes through the restriction point (Pardee, 1974) in G1, after which cells are committed to DNA synthesis to complete the cell cycle and become extracellular growth factor-independent throughout the remainder of the cell cycle (Zetterberg et al., 1995). The retinoblastoma (Rb) protein is the guardian of restriction point (Bartek et al., 1996). When conditions are propitious for cell division without warning signals such as DNA damage or metabolic disturbances, Rb undergoes phosphorylation and become functionally inactivated, permitting the cell to proceed into late G1 (Weinberg, 1995). DNA synthesis takes place in S phase, generating exactly two identical sister chromosomes. G2 phase is a period of rapid cell growth and protein synthesis during which cells get ready for mitosis. DNA damage and replication checkpoint exists in G2 to monitor if chromosome replication is successfully completed and whether DNA damage occurs during the process (Hartwell and Weinert, 1989). This checkpoint determines the fate of a cell, either to enter mitosis or to undergo growth arrest for DNA repair. Mitosis is the process during which eukaryotic sister chromatid get separated to generate two nuclei (Pines and Rieder, 2001). A metaphase checkpoint detects whether all chromosomes are properly attached to mitotic spindles (spindle checkpoint), to ensure equal separation of sister chromatid (Gorbsky, 1995). Cell division is completed by cytokinesis, which segregates the nuclei, cytoplasm, organelles, and cell membrane into two genetically identical daughter cells (Straight and Field, 2000; Glotzer, 2001). Thus, cell cycle is a highly regulated process where multiple checkpoints determine whether to continue or abort a division.

The initiation of specific events during cell cycle, such as preparation for cell division, DNA replication, nuclear membrane breakdown, spindle formation and chromosome segregation, is triggered by a series of protein complexes that are activated in a concerted fashion. The protein complexes, consisting of cyclin and cyclin dependent kinase (CDK), constitute the cell cycle engine, which is driven in a precise sequence with different cyclin-CDK partners activated at specific points of the cell cycle. Mammalian cells accommodate multiple cell cycle-regulatory cyclin and CDKs throughout the cell cycle machinery probably to fine-tune the flexibility of cell cycle control (Nigg, 1995).

After mammalian cells are released from the quiescent state, the first cyclin-CDK holoenzyme known to be activated is composed of a D-type cyclin and either CDK4 or CDK6 depending on the cell type (Matsushime et al., 1994). Cyclin D functions as growth factor sensor, the expression of which is stimulated by growth factor and is independent of the state of the cell cycle (Sherr, 1993). Knowledge of the role of D-type cyclin in G1 progression was obtained from the observation that microinjection of anti-cyclin D1 antibodies into fibroblasts that were in G1 phase prevent them from entering into S phase (Baldin et al., 1993). On the contrary, overexpression of cyclin D1 shortens the duration of G1 phase in mouse fibroblasts, demonstrating cyclin D1 is a rate limiting factor for G1 progression (Quelle et al., 1993). Assembly of cyclin D-CDK4/6 complexes facilitates the phosphorylation of CDK4/6 by a CDK-activating kinase (CAK), which is required for the phosphorylation and enzymatic activation of CDKs (Kato et al., 1994; Kaldis et al., 1998). CAK is composed of three subunits, CDK7, cyclin H and MAT1 (ménage à trois) (Devault et al., 1995). Specifically, phosphorylation of CDKs at Thr161/160/172 by CAK is essential for stabilization of CDK/cyclin complexes by inducing a conformational change that increases the flexibility of the T-loops of all CDKs tested and enables access of the cyclin substrate (Matsuoka et al., 1994; Brown et al., 1999; Morris et al., 2002).

Activated cyclin D-CDK4/6 complexes phosphorylate Rb, which gets further phosphorylated by cyclin E-CDK2 complex at the mid-to-late G1 phase. Hyperphosphorylation inactivates Rb and leads to the release of transcription factor E2F, which activates the transcription of other cell cycle promoting genes, from the blocking effect imposed by hypophosphorylated Rb (Ewen et al., 1993; Weinberg, 1995). From this time on, cell cycle becomes independent of growth factor stimulation for continued cell cycle progression.

Further progression into S phase is driven by cyclin E-CDK2, and thereafter cyclin A-CDK2 promotes the completion of S phase. The precise role cyclin-CDK complexes play in the control of DNA replication is poorly defined. It appears that cyclin E-CDK2 is involved in centrosome duplication (Lacey et al., 1999), and CDK2 is necessary for DNA replication in a cell-free system (Krude et al., 1997). Following DNA synthesis, cells enter into G2 phase and CDK1 is believed to catalyze events in G2/M phase through sequential association with cyclin A and cyclin B. It is reported that the complexing of both cyclin A-CDK2 in late S phase and cyclin A-CDK1 in G2/M phase inactivate anaphase promoting complex/cyclosome (APC/C) to ensure accumulation of key mitotic regulators (Mitra et al., 2006).

Maturation/M phase-promoting factor (MPF) refers to the universal inducer of entry into M phase in eukaryotic cell. Cyclin B-CDK1 is one major component of the MPF complex (Hunt, 1989; Nurse, 1990). Purified cyclin B-CDK1 can induce meiotic G2/M transition upon injection into immature oocytes (Lohka et al., 1988). Proteins that regulate chromosomal condensation, formation of mitotic spindles, and fragmentation of the Golgi apparatus become the substrates of CDK1 during M phase, underscoring the importance of cyclin B-CDK1 complex (Lowe et al., 1998).

Study with CDK or cyclin knockout mice challenged the traditional orderly scheme of cell cycle progression described above. Mice survive in the absence of individual interface CDKs (Rane et al., 1999; Tsutsui et al., 1999; Ye et al., 2001; Berthet et al., 2003; Malumbres et al., 2004), and similar results have been obtained on ablation of cyclin D and cyclin E (Geng et al., 2003; Kozar et al., 2004). It has been shown that CDK4 and CDK6 partner only D-type cyclins, whereas both CDK1 and CDK2 show promiscuity in their choice of cyclin partners and exhibit binding ability with cyclin A, B, D, and E (Aleem et al., 2005; Petri et al., 2007; Santamaría et al., 2007). Thus, a minimal threshold model of cell cycle control has emerged, which suggests that pairing of cyclin A to either CDK1 or CDK2 is sufficient to trigger G1/S transition, whereas cyclin B-CDK1 activity seems to be required for mitosis.

## Cell cycle subversion in viral infection

Viruses keep evolving strategies to subvert the cellular environment of the host for replication and survival. For example, some viral infections induce cell cycle arrest in lymphocytes to inhibit the clonal expansion of anti-viral T or B lymphocytes as a way of immune evasion, whereas carcinogenic viruses are known to antagonize cell cycle checkpoints for neoplastic transformation of infected cells. Thus, elucidating mechanisms of virus-induced deregulation of host cell cycle bears both fundamental and translational relevance.

## Subversion by protein-protein interaction (Table 1, Figure 1)

Normal cellular function including signal transduction and metabolic processes is often mediated through protein-protein interactions, and are routinely targeted by viruses. The host cell cycle is no exception, as illustrated below.

**Table 1:** Cell cycle subversion by protein-protein interaction.

**Virus/Genome**	**Viral protein**	**Host protein**	**Functional consequence**	**References**
HTLV-1/RNA plus	Tax	Cyclin D1/D3	Progression through S phase	Neuveut et al., 1998
HTLV-1/RNA plus	Tax	CDK4	Antagonism to CKI p21^WAF1/CIP1^	Haller et al., 2002
HTLV-1/RNA plus	Tax	p16^INK4A^	Antagonism to p16^INK4A^-imposed blockage of G1 to S transition	Suzuki et al., 1996; Low et al., 1997
HTLV-1/RNA plus	Tax	p15^INK4A^, p15^INK4B^	Restoration of CDK4 activity	Suzuki et al., 1999
HTLV-1/RNA plus	Tax	Chk1, Chk2	Inhibition of kinase activity	Park et al., 2004, 2006
HTLV-1/RNA plus	Tax	DNA-PK	Impairment of the cellular DNA damage response	Durkin et al., 2008
HTLV-1/RNA plus	p30	cyclin E, CDK2	Block of G1/S transition	Baydoun et al., 2010
HIV-1/RNA plus	Tat	p53	Loss of G1/S checkpoint	Clark et al., 2000
HIV-1/RNA plus	Vpr	Wee1, Cdc25C, SLX4, DDB1	G2/M arrest	McGowan and Russell, 1993; Goh et al., 2004; Schröfelbauer et al., 2007; Kamata et al., 2008; Laguette et al., 2014
HBV/circular DNA	HBx	cyclin E/A-CDK2	Stimulation of entry into S phase	Izumiya et al., 2003
HBV/circular DNA	HBx	DDB1	Delayed S phase progression	Leupin et al., 2003; Martin-Lluesma et al., 2008
KSHV/dsDNA	K-bZIP	CDK2, cyclin A, cyclin E	Impairment of CDK2,leading to extended G1 duration	Izumiya et al., 2003
HCV/RNA plus	Core protein	p21^WAF1/CIP1^	Disruption of PCNA-p21^WAF1/CIP1^ binding, impaired cell cycle arrest in G2	Wang et al., 2000
HCV/RNA plus	NS3	p53	Inhibition of oligomerization and transcriptional activation	Deng et al., 2006
HPV/dsDNA	E6	p53	Inhibition of transactivation	Werness et al., 1990; Mietz et al., 1992
HPV/dsDNA	E7	p27^KIP1^	Inhibition of CKI	Zerfass-Thome et al., 1996; Funk et al., 1997; Jones et al., 1997
HPV/dsDNA	E7	Rb	Dissociation of E2F-Rb/p107 complexes	Barbosa et al., 1990; Huang et al., 1993; Wu et al., 1993
Avian Reovirus/dsRNA	p17	CDK1, CDK2, CDK4, CDK6, Cyclin A1, Cyclin D1, Cyclin E1	Cell growth retardation, enhanced replication	Chiu et al., 2018
Influenza A/RNA minus	M2	Cyclin D3	Cell cycle arrest in G0/G1	He et al., 2010; Fan et al., 2017
Influenza A/RNA minus	NS1	RhoA	G-/G1 cell cycle arrest	Jiang et al., 2013
Adenovirus/dsDNA	E1A	p27^KIP1^	Impairment of p27^KIP1^ inhibition on CDK2	Mal et al., 1996
Adenovirus/dsDNA	E1A	Rb	Dissociation of E2F-Rb/p107 complexes	Bagchi et al., 1990; Raychaudhuri et al., 1991; Ikeda and Nevins, 1993
Adenovirus/dsDNA	E1A	p300, CBP	Inhibition of the activation of p21^WAF1/CIP1^, blocking p53-dependent cell cycle arrest	Somasundaram and El-Deiry, 1997
HCMV/dsDNA	IE2-86	CKI	Impaired cell cycle arrest in G2	Sinclair et al., 2000
HCMV/dsDNA	IE2-86, mtrII	p53	Repression of p53 function	Speir et al., 1994; Thompson et al., 1994; Muralidhar et al., 1996; Tsai et al., 1996
SV40/dsDNA	Large T antigen	Rb	Dissociation of E2F-Rb/p107 complexes	Zalvide et al., 1998; Sullivan et al., 2000
SV40/dsDNA	Large T antigen	p53	Inhibition of transactivation	Mietz et al., 1992
Epstein-Barr/dsDNA	EBNA-5, BRLF1	Rb	G0 to G1 progression	Szekely et al., 1993; Zacny et al., 1998
Epstein-Barr/dsDNA	EBNA-3C	Chk2	Reduced Cdc25C phosphorylation, abrogation of the G2/M checkpoint	Choudhuri et al., 2007

**Figure 1:**
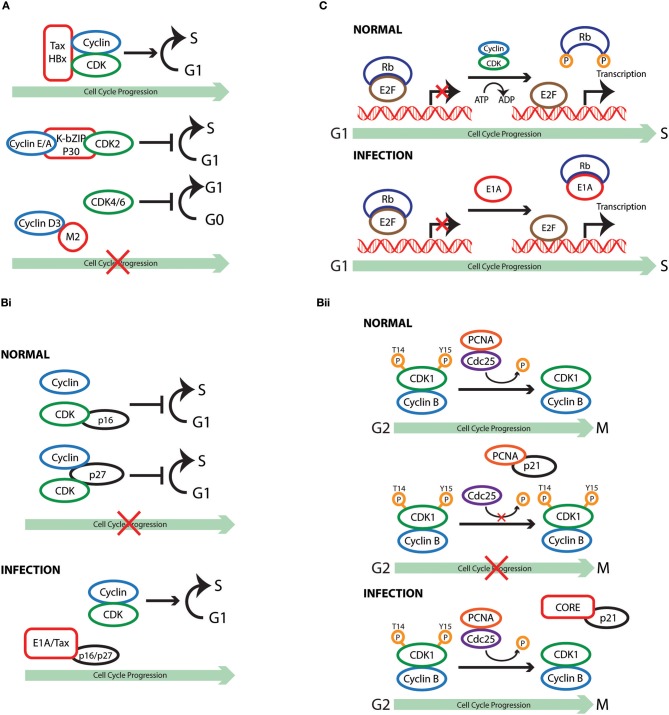
Cell cycle subversion by protein-protein interaction. **(A)** Binding of Tax and HBx proteins to cyclin and/or CDK promotes cell cycle progression either by enhancing kinase activity and/or weakening the inhibitory effect of CKI (Benn and Schneider, 1995; Neuveut et al., 1998; Haller et al., 2002). Direct binding of either K-bZIP protein of KSHV or p30 of HTLV-1 to both CDK2 and cyclin E leads to extended G1 duration or block of G1/S transition (Izumiya et al., 2003; Baydoun et al., 2010). Similarly, direct interaction of M2 of influenza A virus with cyclin D3 arrested cell cycle in G0/G1 phase (Fan et al., 2017). **(Bi)** Binding of E1A to p27^KIP1^ blocks its inhibition on CDK2 kinase activity, overcoming cell cycle arrest in late G1 phase (Mal et al., 1996). In addition to its association with cyclins, HTLV-1 Tax is also able to interact with p16^INK4A^ and relieve p16^INK4A^-imposed blockage of G1 to S transition (Suzuki et al., 1996; Low et al., 1997). **(Bii)** p21^WAF1/CIP1^ can block the interaction between Cdc25C and proliferating cell nuclear antigen (PCNA) by competing with Cdc25C for PCNA binding. This observation points to a role of p21^WAF1/CIP1^ in G2 cell cycle arrest upon DNA damage (Ando et al., 2001). Competition between core protein of hepatitis C virus and PCNA for the association with p21^WAF1/CIP1^ may disrupt PCNA-p21^WAF1/CIP1^ binding, leading to impaired cell cycle arrest in G2 and DNA repair in response to damage signals (Wang et al., 2000). **(C)** E1A protein of adenovirus functions to dissociate E2F-Rb/p107 complexes owing to the interaction of its two conserved regions with Rb (Bagchi et al., 1990; Raychaudhuri et al., 1991). The release of E2F, therefore, transcriptionally activates various downstream target genes that are required for proliferation and DNA synthesis, including c-myc and cyclin E (Roussel et al., 1994; Ohtani et al., 1995).

### Cyclin or CDK

Cyclin and CDK are components that drive the cell cycle clock by forming an active protein complex that could be directly targeted by viruses to modulate cell cycle progression. Binding of the oncoprotein Tax of HTLV-1 to cyclin D1/D3 was observed in transfected cells by co-immunoprecipitation (Neuveut et al., 1998). This interaction is implicated in the ability of Tax to promote progression through S phase, as cells expressing a cyclin D3deficient in Tax-binding exhibit much lesser thymidine incorporation (Neuveut et al., 1998). Interestingly, Haller et al. later demonstrated that Tax could also stimulate CDK4 activity through direct physical interaction via its N-terminal region (Haller et al., 2002). A binding-deficient Tax variant failed to stimulate CDK4-cyclin D complex formation and lost its ability to antagonize the inhibitory effect of cyclin-dependent kinase inhibitor (CKI) p21^WAF1/CIP1^, underlying the importance of protein interaction in Tax-mediated cell cycle regulation (Haller et al., 2002). Chronic hepatitis B virus (HBV) infection is associated with the development of hepatocellular carcinoma, and the X protein of HBV (HBx) was found to stimulate S phase entry of growth-arrested cells (Benn and Schneider, 1995). Co-immunoprecipitation in HBx-expressing cells demonstrated that this viral protein binds to cyclin E/A-CDK2 complexes. Mutant HBx, which displayed disrupted interaction with cyclin-CDK complexes, failed to increase CDK2 activity and, similarly to Tax, lost its effectiveness in counteracting the action ofp27^KIP1^ (Mukherji et al., 2007).

The examples of Tax and Hobs illustrate how binding of viral protein to cyclin or CDK promotes cell cycle progression either by enhancing kinase activity and/or weakening the inhibitory effect of CKI. The interaction of viral protein with cyclin or CDK could also results in hampered cell division. A direct binding of the K-bZIP protein of KSHV to CDK2, cyclin A, or cyclin E has been demonstrated using purified proteins (Izumiya et al., 2003). These interactions were found to impair the kinase activity of CDK2, leading to extended G1 duration, which is accompanied by increased production of the negative regulators p21^WAF1/CIP1^ and p27^KIP1^ (Izumiya et al., 2003). Interestingly p30, another HTLV-1 expressing protein, blocks G1/S transition in contrast to the neoplastic effect of Tax. CyclinE-CDK2 complex formation is hindered in p30 expressing cells due to the interaction of p30 with both cyclin E and CDK2, leading to reduced phosphorylation of the Rb tumor suppressor protein (Baydoun et al., 2010). Infection of influenza A virus (IAV) has been reported to induce host cell cycle G0/G1 phase accumulation (He et al., 2010; Fan et al., 2017). A yeast two-hybrid screen using the cytoplasmic tail of matrix protein 2 (M2) from the highly pathogenic H5N1 strain revealed a high-score interaction with cyclin D3. The M2 ion channel protein is a multifunctional protein with a highly conserved sequence among IAV isolates that approaches 95% identity in some regions (Le and Leluk, 2011; Pielak and Chou, 2011). The physical interaction between M2 and cyclin D3 was confirmed in virus-infected cells, was accompanied by cyclin D3 relocalization and degradation, and resulted in host cell cycle arrest in the G0/G1 phase (Fan et al., 2017). The interaction of viral protein with host cell cycle machinery is not always favorable for viral life cycle. Using a combination of small interfering RNA (siRNA) mediated genetic analysis it has become clear that cyclin D3 restricts IAV production independent of its role in cell cycle. The biogenesis of viral particles involves proper packaging and assembly of viral proteins and RNA to form intact virions at discrete budding sites. M2 is a multifunctional protein: its cytosolic tail interacts with M1, promoting the recruitment of viral internal proteins and viral RNA to the plasma membrane for efficient virus assembly (Chen et al., 2008). The inhibitory effect of cyclin D3 on IAV infection is due to its interaction with viral M2 protein, as measured by competitive co-immunoprecipitation assays, indicating that cyclin D3-M2 interaction either masks the domain on M2 mediating binding to M1, or has a higher affinity than that between M1 and M2. Consequently, limited M1-M2 binding during the budding process of IAV infection results in less progeny virions being efficiently packaged and released from host cells (Fan et al., 2017). These observations suggest a novel function of cyclin D3 that is beyond its classical function in cell cycle regulation. Hence, in this armed-race between cyclin D3 and M2 during IAV infection, the virus evolved strategies to counteract the inhibition imposed by cyclinD3, resulting in hampered cell cycle progression. Most recently, Chiu et al. identified a negatively charged pocket, formed by several conserved acidic residues, which is required for the binding of p17 protein of avian reovirus (ARV) to CDK1 (Chiu et al., 2018). Through GST-pull down assay and *in vitro* kinase assay, the authors showed that p17 competes with cyclin B1 for CDK1 binding and this direct interaction of p17 with CDK1 inhibits the kinase activity of the latter, thus preventing cells from entering mitosis and enhancing virus replication (Chiu et al., 2016, 2018). Unlike its exquisite binding to CDK1 but not cyclin B1, p17 could also suppress CDK2 and CKD4 kinase activities by direct binding to CDKs, partner cyclins, and CDK/cyclin complexes (Chiu et al., 2018).

### CKI

Cyclin-dependent kinase inhibitors are a family of proteins that, acting through separate pathways, determine in cooperation with cyclins and CDKs the decision of the cell to progress through the cell cycle. The adenovirus early region 1A (E1A) protein displays the ability to bind multiple host factors for the manipulation of cell cycle progression. Binding of E1A to p27^KIP1^ blocks its inhibitiononCDK2 kinase activity, overcoming transforming growth factor (TGF)-β-induced cell cycle arrest in late G1 phase (Mal et al., 1996). This provided the first evidence that a viral oncoprotein could manipulate the cell cycle by counteracting an inhibitor of CDKs. In addition to its association with cyclins, HTLV-1 Tax is also able to interact with p16^INK4A^ and relieve p16^INK4A^-imposed blockage of G1 to S transition, demonstrated by the finding that binding-deficient Tax could not protect CDK4 from the inhibitory effect of p16^INK4A^ (Suzuki et al., 1996; Low et al., 1997). Suzuki et al. found that the co-precipitation of p16^INK4A^ by CDK4 was drastically reduced in the presence of Tax, providing direct proof that Tax activates CDK4 by disabling the formation of p16^INK4A^-CDK4 complex (Suzuki et al., 1996). In a similar manner, Tax restores CDK4 activity through interaction with p15^INK4B^, further contributing to the immortalization of T cells (Suzuki et al., 1999). Interestingly, while Tax directly antagonizes p16^INK4A^ and p15^INK4B^ through protein-protein interaction, it utilizes a different mechanism to repress p18^INK4C^ transcription and further promote cell transformation (Suzuki et al., 1999).

It was reported that p21^WAF1/CIP1^ could block the interaction between cell division cycle 25C (Cdc25C), a phosphatase of CDK1 that activates the CDK1/cyclin B1 complex (Strausfeld et al., 1991), and proliferating cell nuclear antigen (PCNA) by competing with Cdc25C for PCNA binding. This observation points to a role of p21^WAF1/CIP1^ in G2 cell cycle arrest upon DNA damage (Ando et al., 2001). Formation of a complex between the core protein of hepatitis C virus (HCV), which plays an important role in the development of hepatocellular carcinomas, and p21^WAF1/CIP1^ was mapped to the C-terminus of this CKI, a region in the close vicinity of the PCNA binding site of p21^WAF1/CIP1^ (Wang et al., 2000). *In vitro* experiments revealed that a competition exists between core protein and PCNA for the association with p21^WAF1/CIP1^ (Wang et al., 2000). It is plausible to speculate that, in the case of HCV infection, expression of core protein may disrupt PCNA-p21^WAF1/CIP1^ binding, leading to impaired cell cycle arrest in G2 and DNA repair in response to damage signals. Other examples of viral protein-mediated inhibition of CKIs include the E7 oncoprotein of HPV-16 and the immediate-early (IE) protein IE2-86 of human cytomegalovirus (HCMV) (Zerfass-Thome et al., 1996; Funk et al., 1997; Jones et al., 1997; Sinclair et al., 2000).

### Rb/pocket proteins or E2Fs

Growth factors are necessary to drive the cell cycle machinery to the restriction point, beyond which commitment to cell cycle progression occurs and the cell enters S phase without requiring extracellular mitogenic signals. Retinoblastoma (Rb) protein, which serves as the guardian of restriction point, represents a non-redundant checkpoint that can be targeted by viruses to modulate host cell cycle under growth limiting conditions (Blagosklonny and Pardee, 2002). For example, the adenovirus E1A not only associates with CKI, but also functions to dissociate E2F-Rb/p107 complexes owing to the interaction of its two conserved regions (CRs) with Rb (Bagchi et al., 1990; Raychaudhuri et al., 1991). It was proposed that the LXCXE motif-containing CR2 of E1A mediates its initial binding to Rb, allowing CR1 to block the sequences on Rb that are involved in E2F recognition by means of physical interaction (Ikeda and Nevins, 1993). The release of E2F, therefore, transcriptionally activates various downstream target genes that are required for proliferation and DNA synthesis, including c-myc and cyclin E (Roussel et al., 1994; Ohtani et al., 1995). Similarly, HPV E7 possesses two homology regions of CR1 and CR2, which contribute to the transformation properties of E7 through disruption of E2F-Rb complexes (Barbosa et al., 1990; Huang et al., 1993; Wu et al., 1993). The association of the SV40 large T-antigen (L-Tag), which is expressed early during infection and is essential for viral replication, with Rb, p107, and p130 is attributed to the viral LXCXE residues, which are required but not sufficient in L-Tag-mediated transformation (DeCaprio et al., 1988; Chen and Paucha, 1990; Thompson et al., 1990; Stubdal et al., 1996). It was reported that the N-terminus of L-Tag, which shares sequence homology with the J domain of heat shock protein 40 (HSP40), is also required to disrupt the complex formation between the three “pocket” proteins of the Rb family with E2F (Zalvide et al., 1998; Sullivan et al., 2000).

The nuclear antigen 5 (EBNA-5), also known as nuclear antigen leader protein (EBNA-LP), of Epstein-Barr virus (EBV) cooperates with its nuclear antigen 2 (EBNA-2) to prompt G0 to G1 progression during the immortalization of virus-infected cells (Sinclair et al., 1994). Unlike the E1A of adenovirus, E7 of HPV, and L-Tag of SV40, EBNA-5 localizes with and binds to Rb without possessing a LXCXE motif. Although study revealed that the carboxy-terminal 45 amino acids of EBNA-5 are crucial for B cell transformation (Jiang et al., 1991; Mannick et al., 1991; Szekely et al., 1993), the Rb binding region on EBNA-5 was mapped to a 66-amino acid-long peptide that locates on the N-terminal half of this viral protein, which is also the binding site for p53 (Szekely et al., 1993). It was found that p53 competes with Rb for EBNA-5 binding as it inhibits EBNA-5-Rb complex formation *in vitro* in a dose-dependent manner. However, inhibition of p53-EBNA-5 interaction by Rb was not observed in a reciprocal experiment (Szekely et al., 1993). The fact that the transformation-capable region on EBNA-5 is distinct from its Rb/p53-binding motif suggests interaction with Rb/p53 is not related to its ability to induce B cell transformation. Indeed, Inman et al. showed that expression of EBNA-5 could neither relieve Rb-mediated repression of E2F1 transactivation, nor prevent p53-induced transactivation (Inman and Farrell, 1995). However, it is possible that the association of EBNA-5 to Rb affects other aspects of its function, such as its control of c-Abl tyrosine kinase, to aid in viral infection. In addition, Zancy et al. reported an interaction of EBV immediate-early lytic gene product BRLF1 with Rb in virus-infected cells (Zacny et al., 1998). Although the interaction motif on Rb for BRLF1 binding is outside of its pocket region and BRLF1 does not interact with E2F, a correlation was still observed between the kinetics of BRLF1-Rb binding and the displacement of E2F1 from Rb, suggesting the potential of BRLF1 in initiating cell cycle progression (Zacny et al., 1998).

### Chk

Checkpoint kinase (Chk) 1 and 2 are tumor repressor proteins that are activated upon replication defect and/or DNA damage (Bartek and Lukas, 2003; Haoudi et al., 2003; Park et al., 2004, 2006). Being a multifunctional viral protein, Tax of HTLV-1 is also known to interact with Chk. Thus, expression of Tax inhibits the kinase activity of Chk1, whereas the effect of Tax on the kinase activity of Chk2 is controversial. Park et al. showed that Cdc25A degradation and p53 phosphorylation (Ser20), the downstream events of Chk1 activation, were attenuated by Tax expression in a dose-dependent manner, suggesting that Tax inactivates Chk1 (Hirao et al., 2000; Shieh et al., 2000; Xiao et al., 2003; Park et al., 2004). Two domains on Tax were identified to be important for both binding to and inhibition of Chk2, drawing a causal relationship between physical protein interaction and the functional inactivation of Chk family of tumor repressors (Park et al., 2006). By contrast, Haoudi et al. described an induction of the steady-state level of Chk2, but not Chk1, by Tax with increased Cdc25C degradation and p53 phosphorylation (Ser15) (Haoudi et al., 2003). Despite the fact that Chk2 was revealed to be activated by Tax, the overall response of Chk2 to DNA damage was impaired through inhibiting the release of Chk2 from chromatin, which is normally observed upon exposure to ionizing radiation (Li and Stern, 2005a; Gupta et al., 2007). This discrepancy in the regulatory role of Tax on Chk2 activity may be explained by the differences in the assay systems adopted by these studies. Alternatively, it may reflect the dual action of Tax during different phases of the same event, as Tax could first induce the accumulation of Cdc25C, which undergoes degradation at a later stage (Haoudi et al., 2003). However, regardless of whether Tax activates or inhibits the activity of Chk2, the cellular DNA damage response mediated by Chk2 is dampened. In addition, an association of Tax with the DNA-dependent protein kinase (DNA-PK), an early mediator of cell cycle arrest via the activation of Chk2, has also been reported (Li and Stern, 2005b; Durkin et al., 2008). Durkin et al. observed increased DNA-PK phosphorylation, an initiating event required for the activation of the kinase activity of DNA-PK (Li and Stern, 2005b), in Tax-expressing cells (Durkin et al., 2008). Tax-induced constitutive signaling of the DNA-PK pathway, however, impairs cellular response to new damage (Durkin et al., 2008). Thus, through saturating DNA-PK-mediated damage repair response, Tax inactivates Chk2. Furthermore, the delay and impairment of the cellular DNA damage response imposed by Tax appears to be regulated by molecular sequestration of DNA-PK by Tax through physical interaction (Durkin et al., 2008). The nuclear antigen 3C (EBNA-3C) of Bevies is another viral protein that interacts with Chk2, resulting in reduced Cdc25C phosphorylation at Ser216. This Chk2-dependent step causes sequestration of Cdc25C in the cytoplasm, which prevents removal of the inhibitory phosphates from CDKs, leading to EBNA3C-mediated abrogation of the G2/M checkpoint and transformation in lymphoblastoid cell lines (Choudhuri et al., 2007).

### P53

The tumor suppressor, p53, was first identified as a host protein that stably interacts with the L-Tag of SV40 (Lane and Crawford, 1979; Linzer and Levine, 1979; Tan et al., 1986). The use of chloramphenicol (CAT) assay demonstrated that L-Tag inhibits the transactivation function of p53, which is mediated through physical interaction as L-Tag mutants deficient in p53 binding fail to prevent downstream activation of gene expression (Mietz et al., 1992). Similarly, complex formation between HPV E6, limited only to the oncogenic HPV types (Werness et al., 1990; Mietz et al., 1992), and p53 leads to a drastic reduction (more than 80%) in the level of p53-mediated transactivation in CAT assay system. HCMV IE2-86 is able to repress p53 function via physical interaction and this inhibitory effect requires two functional domains on IE2-86 (Speir et al., 1994; Tsai et al., 1996). Tsai et al. showed that the N-terminus of IE2-86 was necessary for p53-binding while the C-terminal domain was needed to inhibit p53-mediated transactivation, suggesting that IE2-86 is recruited by protein interaction to target p53 and exert its inhibitory effect (Tsai et al., 1996). Another HCMV protein, which alters p53-orchestrated cellular regulatory mechanisms, leading to tumorigenic transformation in rodent cells, is the morphological transforming region II (mtrII) (Thompson et al., 1994; Muralidhar et al., 1996). It was reported that the first 49 amino acids of mtrII mediate its binding to p53 and are sufficient to repress p53-activated transcription (Muralidhar et al., 1996).

Binding of the nonstructural protein 3 (NS3) of HCV to p53 has been located to the C-terminus, a region that also contains the oligomerization domains required for the latter to form the bioactive homo-tetramer (Levine, 1997; Ishido and Hotta, 1998). It has been proposed that binding of NS3 to p53 interferes with the formation of p53 tetramer, which is most effective in transactivating its target genes, leading to impaired p53-dependent transcriptional activation (Deng et al., 2006). Interestingly, other than binding to p53 itself, adenovirus E1A targets p53-mediated transcription through interacting with the transcriptional coactivators p300 and CREB-binding protein (CBP) of p53 (Somasundaram and El-Deiry, 1997). As a result, E1A inhibits the activation of p21^WAF1/CIP1^, abolishing p53-dependent cell cycle arrest. An interaction between the transactivator of transcription (Tat) of HIV-1 and p53 was correlated to the development of acquired immune deficiency syndrome (AIDS)-related malignancies. Thus, Clark et al. found that the presence of Tat almost completely abolished p53-mediated activation, leading to reduced levels of p21^CIP1/WAF1^ and the loss of G1/S checkpoint (Clark et al., 2000). However, it remains to be addressed whether a causal relationship exists between Tat-p53 interaction and the repression of p53 function, which will require the use of p53 binding-deficient Tat.

### Other factors

Interactions of viral component with other cell-cycle regulators have been reported for several viruses. For example, Jiang et al. have shown that expression of influenza A virus nonstructural protein 1 (NS1) downregulates the protein level and inhibits the function of the Ras homolog gene family member A (RhoA), a small GTPase that, besides its primary involvement with formation of actin stress fibers and cytoskeletal remodeling, mediates cell cycle G1/S transition (Jiang et al., 2013). The inhibition of RhoA activity by NS1 was reported to be achieved through physical interaction between the two proteins.

Wee1, a protein kinase which inactivates CDK1 by phosphorylating the latter on Tyr15, can interact with HIV-1 viral protein R (Vpr) in cell culture, resulting in enhanced kinase activity of Wee1 (McGowan and Russell, 1993; Kamata et al., 2008). Although this interaction is required,it is not sufficient for the induction of cell cycle arrest as Vpr mutants, which are unable to induce cell cycle arrest, maintain their binding and activation to Wee1, suggesting the existence of additional factors in Vpr-mediated activation of the G2 checkpoint (Kamata et al., 2008). It is interesting to note that Wee1 blocks G2toM transition by phosphorylating CDK1, whereas Cdc25 triggers this transition and entry into mitosis by dephosphorylating the same residues on CDK1 (Millar and Russell, 1992). In addition to its binding with Wee1, Vpr was also shown to associate with Cdc25C. This binding was mapped to a site that lies near but is distinct from the catalytic domain of Cdc25C (Goh et al., 2004). In accordance with its activation of Wee1, Vpr inhibits the activity of Cdc25C, most likely through its ability to bind Cdc25C (Goh et al., 2004). Thus, Vpr targets CDK1 to mediate G2/M arrest acting on both Wee1 and Cdc25C.

Furthermore, several groups recently demonstrated a direct interaction of Vpr withSLX4, a scaffold for the formation of a heterodimeric structure-specific endonuclease consisted of MUS81 and EME1 (Fekairi et al., 2009; Muñoz et al., 2009; Svendsen et al., 2009; Kim et al., 2013; Laguette et al., 2014). Thus, binding of Vpr to SLX4 activates the MUS81-EME1 endonuclease, leading to replication stress. The observation that G2/M arrest-deficient Vpr mutants fail to interact with SLX4, and that silencing of SLX4 reduces Vpr-dependent cell cycle perturbation highlight the significance of Vpr-SLX4 association in Vpr-induced G2/M arrest (Laguette et al., 2014). In accordance, the use of a panel of simian immunodeficiency virus (SIV) Vprs that differ in their ability to promote cell cycle arrest demonstrated the correlation between Vpr-SLX4binding and the G2/M arrest effect of Vpr (Berger et al., 2015).

DNA damage-binding (DDB) protein, which constitutesDDB1 and DDB2 subunits, translocates to the nucleus to mediate DNA repair in response to DNA damage signal (Wittschieben and Wood, 2003; Iovine et al., 2011). The formation of a complex comprising HIV-1 Vpr, DDB1-Cullin 4A-associated factor 1 (DCAF1), and DDB1 has been implicated as the upstream event in Vpr-triggered G2/M arrest (Le Rouzic et al., 2007; Schröfelbauer et al., 2007). It is known that Vpr hampers the nuclear localization of DDB1, and interferes with the binding of DDB1 to damaged DNA as detected by an electrophoretic mobility shift assay (EMSA) (Schröfelbauer et al., 2007). Moreover, the association of Vpr with DDB1 disrupts complex formation between DDB1 and DDB2, which is a prerequisite for DNA repair (Schröfelbauer et al., 2007). The significance of Vpr-DDB1 interaction in Vpr-mediated cytostatic effect was revealed through the use of DDB1 binding-deficient Vpr mutant (Schröfelbauer et al., 2007). It is worth mentioning that one of the multiple functions of DDB1 is to act as a component of an ubiquitin-E3 ligase complex that targets cell cycle regulatory proteins such as p27^KIP1^ for proteasomal degradation (Bondar et al., 2006). It will be of interests to investigate whether the association of Vpr with DDB1 could antagonize its role in the hydrolysis of p27^KIP1^ and other cell cycle effectors, thus contributing to cell cycle arrest. Indirect support for this hypothesis stems from experiments with transient expression of HBV HBx, which induces accumulation of lagging chromosomes and other mitotic aberrations, leading to the activation of DNA replication checkpoint with a delayed S phase progression (Martin-Lluesma et al., 2008). The use of an HBx mutant that is deficient for DDB1 binding suggests that this perturbation of cell cycle progression is attributable to the interaction of HBx with DDB1 (Leupin et al., 2003; Martin-Lluesma et al., 2008).

## Subversion by protein phosphorylation (Table 2 and Figure 2)

Phosphorylation and dephosphorylation govern the function of many cell cycle regulators, thus acting as switches to ensure its normal progression. For instance, the kinase activity of CDK4 cannot be merely regulated by its association with D-type cyclin. CDK4 phosphorylation onThr172 by CAK is also required to catalyze the process (Kato et al., 1994). As another example, the inhibitory effect of Rb on transcription factor E2F1is suppressed through CDK4/6-mediated hyperphosphorylation of Rb (Sidle et al., 1996). Not surprisingly, viruses have evolved mechanisms to modulate phosphorylation and dephosphorylation events as additional means to subvert host cell division cycle for their own benefit.

**Table 2:** Cell cycle subversion by protein phosphorylation.

**Virus/Genome**	**Viral protein**	**Host protein**	**Functional consequence**	**References**
HIV-1/RNA plus	Vpr	Phosphorylation of Chk1	G2/M arrest	Li et al., 2010
RVFV/RNA minus	NS proteins	Phosphorylation of ATM and Chk2	S phase arrest	Baer et al., 2012
HBV/circular DNA		Phosphorylation of Chk1 and p53	Transient cell cycle arrest in S and G2 phases	Zhao et al., 2008
HSV-1/dsDNA	ICP0	Phosphorylation of ATM and Chk2	CDK1 inactivation with ensuing G2/M arrest	Ahn et al., 2000; Li et al., 2008
HHV-6/dsDNA	UL24 family protein	Phosphorylation of CDK1	G2/M arrest	Nascimento et al., 2009; Li et al., 2011
Reovirus/dsRNA	sS1	Phosphorylation of CDK1	G2/M arrest	Poggioli et al., 2000, 2001
HCMV/dsDNA	IE1-72	Phosphorylation of E2Fs, p107, p130	Transcriptional activation, cell cycle progression	Pajovic et al., 1997
HCMV/dsDNA	UL97	Phosphorylation of Rab	Phosphorylation and inactivation of Rab	Hume et al., 2008

**Figure 2:**
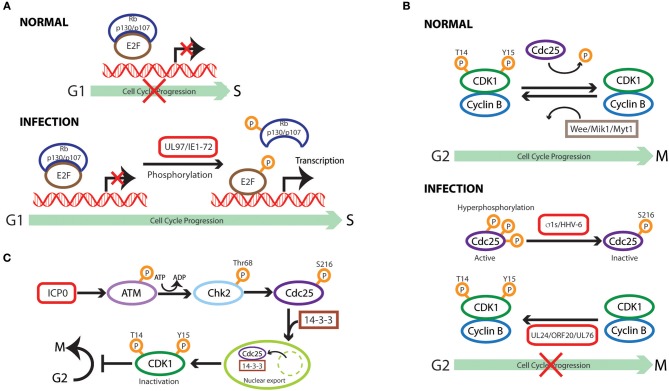
Cell cycle subversion by protein phosphorylation. **(A)** Direct phosphorylation of Rb by HCMV UL97 protein dissociates E2F-Rb/p107 complexes releasing E2F to activate transcription and cell cycle progression (Hume et al., 2008). HCMV IE1-72 is a viral protein kinase able to directly and selectively phosphorylate transcription factors of the E2F family (E2F1,−2, and−3), leading to E2F-dependent transcriptional activation, thus regulating cell cycle events (Pajovic et al., 1997). **(B)** Expression of viral S1 gene-encoded σ1s nonstructural protein during reovirus infection leads to G2/M arrest in host cells via σ1s-mediated CDK1 hyperphosphorylation (Poggioli et al., 2000, 2001). Similarly, human herpesvirus 6 (HHV-6)-induces G2/M arrest by increasing the inactive Ser216-phosphorylated form Cdc25C phosphatase that accumulates in the cytoplasm where it has no access to its CDK1 substrate (Li et al., 2011). Members of herpesvirus share the conserved UL24 family of proteins (i.e., UL24, ORF20, and UL76), whose expression induces CDK1 phosphorylation at Tyr15 inhibitory site with ensuing cell cycle G2/M arrest (Nascimento et al., 2009). **(C)** HSV-1 immediate-early gene product ICP0 triggers a series of phosphorylation events resulting in cytoplasmic sequestration of Cdc25C, which maintains high levels of CDK1 inhibitory phosphorylation, leading to CDK1 inactivation with ensuing G2/M arrest (Li et al., 2008).

### Viral kinases

Direct phosphorylation of Rb was reported for HCMV UL97 protein as the first example of a virus-encoded functional ortholog of CDK (Hume et al., 2008). As expected, expression of mutant UL97 in cells, which are intrinsically deficient in the ability to phosphorylate Rb, induces Rb phosphorylation on the inactivating residues. Moreover, UL97 rescues the proliferation of a yeast mutant which lacks CDK activity, suggesting UL97 functions similar to CDK. Sequence comparison between CDK and UL97 revealed that this viral protein does not require cyclin binding for kinase activity and, therefore, is resistant to the inhibition of p21^WAF1/CIP1^, features which shield UL97 from the normal cellular control mechanisms that could attenuate CDK activity (Hume et al., 2008).

HCMV IE1-72 is a viral protein kinase, which is able to directly and selectively phosphorylate transcription factors of the E2F family (E2F1,−2, and−3) and pocket proteins (p107 and p130) (Pajovic et al., 1997). Its kinase activity is required for IE1-72-mediatedformation and/or dissociation of inhibitory complexes that lead to E2F-dependent transcriptional activation, thus regulating cell cycle events (Pajovic et al., 1997). It is interesting to note that the kinase activity of IE1-72 resides in the portion encoded by exon 4, which is not the same region necessary for its binding to p107 (encoded by exon 3) as mentioned above (Johnson et al., 1999). It is possible that this interaction functions as the first step to bridge IE1-72 to its target for later phosphorylation, or that IE1-72 can independently exert its role in cell cycle regulation through either physical interaction or its kinase activity.

### CDK1/Cdc25

Reovirus infection and the sole expression of viral S1 gene-encoded σ1s nonstructural protein could lead to G2/M arrest in host celosia σ1s-mediated CDK1 hyperphosphorylation as revealed with the use of σ1s-mutant virus (Poggioli et al., 2000, 2001). It was reported that the inhibitory phosphorylation of CDK1 by reovirus decreased the hyperphosphorylated form (active form) of Cdc25C, the CDK1-specific phosphatase (Poggioli et al., 2001), hence preventing CDK1 dephosphorylation, an absolute requirement for the onset of mitosis (Jin et al., 1996). Similarly, human herpesvirus 6 (HHV-6)-induced G2/M arrest correlates with enhanced phosphorylation of CDK1 at the Tyr15 inhibitory site. This is achieved through both elevating the level of the negative regulator Wee1 and inhibiting the activity of Cdc25C phosphatase by increasing the inactive Ser216-phosphorylated form that accumulates in the cytoplasm where it has no access to its CDK1 substrate (Li et al., 2011). Members of herpesvirus share the conserved UL24 family of proteins, namely UL24 in herpes simplex virus type 1 (HSV-1), ORF20 in KSHV, and UL76 in HCMV. Expression of these UL24 proteins led to CDK1 phosphorylation at Tyr15, the dephosphorylation of which is required for the initiation of mitosis (Jin et al., 1996), with ensuing cell cycle G2/M arrest (Nascimento et al., 2009). Similar observations were made with murine gammaherpesvirus 68 (MHV68) infection (Nascimento and Parkhouse, 2007).

The expression of HSV-1 immediate-early gene product ICP0 in HEK 293T cells activates Chk2, but not Chk1, through Thr68 phosphorylation (Li et al., 2008). This activation relies on ICP0-dependent phosphorylation of ataxia telangiectasia mutated (ATM), an upstream effector kinase of Chk2 (Ahn et al., 2000), as revealed in ATM-deficient cell lines (Li et al., 2008). Activated Chk2 in turn phosphorylates Cdc25 on Ser216, which promotes binding of the molecule to 14-3-3 proteins, resulting in cytoplasmic sequestration of Cdc25C, as discussed above, which maintains high levels of CDK1 inhibitory phosphorylation (Matsuoka et al., 1998; Graves et al., 2001). The requirement for the kinase activity of Chk2 in ICP0-induced inhibitory-phosphorylation of Cdc25C was demonstrated by reconstituting Chk2-deficient cells with kinase-defective mutant form of Chk2 (Li et al., 2008). Thus, through serial phosphorylation events, ICP0 leads to CDK1 inactivation with ensuing G2/M arrest. Similarly, ATM and Chk2 phosphorylation are required for Rift Valley fever virus (RVFV)-induced S phase arrest, a phenomenon attributed to the activity of viral nonstructural proteins (Baer et al., 2012).

Infection of a hepatocyte cell line with HBV causes phosphorylation of Chk1 and p53at Ser345 and Ser15respectively, which is accompanied by a transient cell cycle arrest in S and G2 phases (Zhao et al., 2008). Although more remains to be done to elucidate the signaling pathway concerning these phosphorylation events, it is known that Chk1 phosphorylation on Ser345 is essential for the activation of checkpoint arrest in G2/M phase (Lopez-Girona et al., 2001). Similarly, Chk1 phosphorylation at Ser345 is required in G2/M arrest induced by HIV-1 Vpr as re-introduction of a phosphorylation-resistant Chk1 mutant fails to restore Vpr-induced G2/M arrest in Chk1-depleted cells (Li et al., 2010). Thus, by phosphorylating Chk1, Vpr facilitates proteasome-mediated degradation of Cdc25C and, to a lesser extent, Cdc25B, contributing to G2/M arrest (Lopez-Girona et al., 1999; Graves et al., 2001; Bulavin et al., 2003).

### CDK2

CDKs remain catalytically inactive until cyclin-bound CDKs undergo phosphorylation at Thr161/160/172 by CAK (Gould et al., 1991; Norbury et al., 1991; Desai et al., 1992; Gu et al., 1992; Krek and Nigg, 1992; Solomon et al., 1992). The current model of CDK/cyclin activation posits two steps: (i) formation of an intermediate complex composed by the PSTAIRE helix of CDK2 and helices 3 and 5 of the cyclin; (ii) isomerization of the CDK/cyclin complex to expose the T-loop of CDK, which is then accessible to phosphorylation by CAK to form the substrate binding site (Morris et al., 2002). CDK2/CDK4 phosphorylation at their respective Thr sites by CAK subunits, for example CDK7/cyclin H (Fisher and Morgan, 1994), is essential for both the stabilization of CDK/cyclin complexes and could increase the flexibility of the CDK2 T-loop (Matsuoka et al., 1994; Morris et al., 2002). Overexpression of the ARV p17 protein inhibited CDK2 phosphorylation at T160, which was dependent on CDK7/cyclin H disassociation due to increased p53/cyclin H interaction (Chiu et al., 2018). Thus, by diminishing the CAK activity, namely CDK7/cyclin H association, p17 prevents CDK2 phosphorylation and therefore inhibits its kinase activity. Although the mechanism of how p17 diminishes CAK activity in a p53-dependent manner remains to be elucidated, it is another example illustrating how viruses regulate the cell cycle machinery (Chiu et al., 2018).

## Subversion by protein degradation (Table 3 and Figure 3)

Proteolysis of cell cycle regulators at defined stages plays a crucial role in cell cycle progression. While purified recombinant IE2-86 of HCMV is able to block the inhibitory effect of the cyclin-dependent kinase inhibitor p21^WAF1/CIP1^ through physical interaction as previously mentioned, HCMV infection can also target p21^WAF1/CIP1^ todegradation. Thus, a disparity between p21^WAF1/CIP1^ RNA and protein levels was observed after HCMV infection, implying possible degradation of p21^WAF1/CIP1^ in virus-infected cells (Chen et al., 2001). Infection could induce sustained increase in calpain activity and the use of specific inhibitors demonstrated that calpain, and not proteasome activity is required for HCMV-mediated p21^WAF1/CIP1^ reduction, although the molecular mechanism by which p21^WAF1/CIP1^ is targeted by calpain is not clear (Chen et al., 2001).

**Table 3:** Cell cycle subversion by protein degradation.

**Virus/Genome**	**Viral protein**	**Host protein**	**Functional consequence**	**References**
HTLV-1/RNA plus	Tax	Association with accessory factor CDC20 of APC	Activation of anaphase promoting complex	Liu et al., 2005
HTLV-1/RNA plus	Tax	Polyubiquitination and degradation of Skp2	Stabilization of p21^WAF1/CIP1^ and p27^KIP1^ CKIs, cell cycle G1 arrest	Carrano et al., 1999; Nakayama et al., 2004; Kuo and Giam, 2006
HIV-1/RNA plus	Vif	Inhibition of p53 ubiquitination and degradation	Cell cycle arrest in G2	Izumi et al., 2010
HBV/circular DNA	HBx	Upregulation of USP37 transcripts	Accumulation of cyclin A and acceleration of the G1/S phase transition	Saxena and Kumar, 2014
HBV/circular DNA	HBx	Reduction of Notch cleavage	Induction of G1-S cell cycle progression	Wang et al., 2010; Xu et al., 2010
HSV-1/dsDNA	ICP0	CENP-C, CENP-A proteasome degradation	Mitotic block	Everett et al., 1999; Lomonte et al., 2001
Japanese Encephalitis virus/RNA plus		Degradation of p21^WAF1/CIP1^ and p27^KIP1^ CKIs	Bypass G0/G1 arrest	Das and Basu, 2008
EV71/RNA plus		Cyclin A2 proteasome degradation	Prevent cell cycle transition from S to G2/M phase	Ooi et al., 2007; Yu et al., 2015
Influenza A/RNA minus	M2	Cyclin D3	Cell cycle arrest in G0/G1	Fan et al., 2017
Influenza A/RNA minus		Nucleoprotein (NP)	Inhibits MDM2-mediated p53 degradation; apoptosis	Wang et al., 2012
HCMV/dsDNA		Calpain-dependent p21^WAF1/CIP1^ degradation	Impaired cell cycle arrest in G2	Chen et al., 2001
Avian Reovirus/dsRNA	P17	Phosphorylation of p53	Prevents MDM2-mediated p53 degradation; cell cycle arrest	Lin et al., 2009

**Figure 3:**
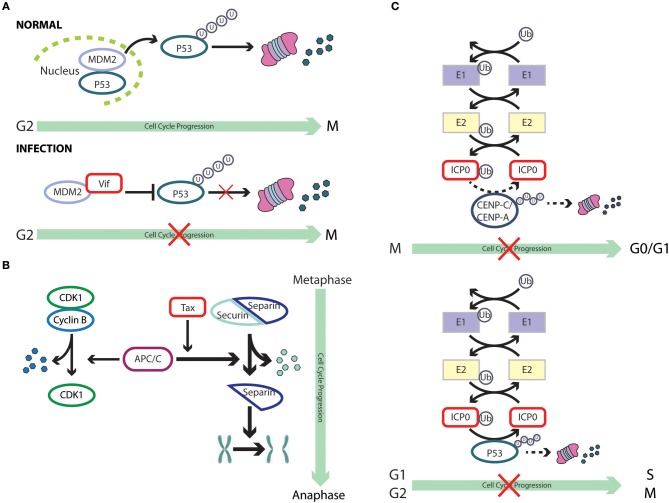
Cell cycle subversion by protein degradation. **(A)** Expression of (Vif) protein of HIV-1 blocks MDM2-mediated proteasomal degradation of p53, by binding to both MDM2 and p53, leading to cell cycle arrest in G2 (Izumi et al., 2010). **(B)** The degradation of securin by the anaphase promoting complex/cyclosome (APC/C) is essential for the completion of mitosis, leading to correct transmission of chromosomes from mother to daughter cells. The oncoprotein Tax of HTLV-1 is able to promote securin and cyclin B1 degradation, leading to chromosome instability (Liu et al., 2003). **(C)** ICP0 of HSV-1 possesses an ubiquitin E3 ligase activity (Boutell et al., 2002; Hagglund et al., 2002), which results in rapid loss of the centromeric protein CENP-C and CENP-A in a proteasome-dependent manner and mitotic block (Everett et al., 1999; Lomonte et al., 2001). However, it remains unclear whether these two centromere components are direct substrates of the ICP0 E3 ligase (dashed line). Likewise, ICP0 can directly ubiquitinate p53 (Boutell and Everett, 2003), although p53 levels are not reduced during HSV-1 infection (Hobbs and DeLuca, 1999) and, hence, the role of p53 in ICP0-induced perturbation of cell cycle progression remains unclear (dashed line).

The interaction between cyclin D3 and M2 during Influenza A infection provides another example of the involvement of the proteasomal degradation pathway. Although the interaction was found to inhibit virus production, this mode of restriction eventually surrenders to viral infection as cyclin D3 becomes trapped in the cytoplasm by M2 and eventually undergoes proteasomal degradation upon prolonged infection (Fan et al., 2017). Indeed, mislocalization and subsequent downregulation of restriction factors is one of the recurring mechanisms viruses have adopted to overcome host defenses (Ma et al., 2012). In the case of IAV, two possible mechanisms account for the relocalization of cyclin D3 to the cytoplasm: (i) M2 interacts with newly synthesized cyclin D3 and prevents it from translocating into the nucleus, or (ii) cyclin D3 is retained by M2 in the cytoplasm after its nuclear export. It will be interesting to explore these mechanisms in greater detail in future studies.

HIV-1 viral infectivity factor (Vif) binds to the central domain of mouse double-minute 2 protein (MDM2), the primary E3 ubiquitin ligase for p53 (Momand et al., 2000; Izumi et al., 2009). Izumi et al. showed that expression of Vif could block MDM2-mediated proteasomal degradation of p53, leading to cell cycle arrest in G2 (Izumi et al., 2010). More interestingly, the same study reported that Vif could directly interact with p53, independent of its association with MDM2, leading to a partial blockage of p53-MDM2 binding and, hence, impairment of MDM2-mediated p53 degradation. This strategy appears to be very effective and economical, as it has been adopted by other viruses. For example, the IAV nucleoprotein (NP) binds to p53 with ensuing protein stabilization as a result of compromised MDM2-mediated p53 degradation (Wang et al., 2012). ARV infection promotes p53 phosphorylation at multiple sites, an event which alleviates p53 inhibition by MDM2 (Shieh et al., 1997), and activates the transcriptional function of p53 (Lin et al., 2009). It was later discovered that p17 protein of ARV could robustly enhance MDM2 binding to ribosomal proteins, thereby blocking MDM2-mediated p53 ubiquitination and degradation (Zhang and Lu, 2009; Huang et al., 2017). Thus, through multiple mechanisms, ARV impairs the ability of MDM2 to target p53 for degradation. There is evidence that both PTEN and p21^WAF1/CIP1^ are required for p53-mediated cell cycle arrest (el-Deiry et al., 1993; Stambolic et al., 2001), which is dependent on a complex signaling cascade that brings about Akt-induced phosphorylation of MDM2, antagonized by PTEN, and the ensuing translocation of MDM2 to the nucleus to downregulate p53 (Mayo and Donner, 2002). Thus, the interplay between viruses and tumor suppressor proteins is likely to target multiple networks that ultimately affect the cell cycle.

The anaphase promoting complex/cyclosome (APC/C) was characterized as a securin- and cyclin B-specific ubiquitin ligase, functioning mainly to trigger metaphase to anaphase transition by tagging these proteins for degradation (Irniger et al., 1995; King et al., 1995; Sudakin et al., 1995; Thornton and Toczyski, 2003; Peters, 2006). Securin sequesters separin, the “sister-separating” protease, preventing the separation and segregation of sister chromatids (Nasmyth et al., 2000; Yanagida, 2000). Thus, the degradation of securin by APC/C is essential for the completion of mitosis, leading to correct transmission of chromosomes from mother to daughter cells (Zou et al., 1999). The oncoprotein Tax of HTLV-1 is able to promote securin and cyclin B1 degradation, a process which requires the function of the accessory factor CDC20 of APC, leading to chromosome instability (Liu et al., 2003). Although the mechanism by which Tax activates APC is still unclear, an association of Tax with APC^CDC20^ was reported (Liu et al., 2005). Interestingly, premature activation of APC by Tax could also result in a surge in the levels of p21^WAF1/CIP1^ and p27^KIP1^, leading to G1 arrest that resembles cellular senescence (Kuo and Giam, 2006). This was found to be a result of polyubiquitination and degradation of Skp2, the substrate recognition subunit of the SCF^Skp2^ E3 ubiquitin ligase that regulates the destruction of p21^WAF1/CIP1^ and p27^KIP1^, by Tax-activated APC (Carrano et al., 1999; Nakayama et al., 2004; Kuo and Giam, 2006). Thus, by targeting the E3 ligase of p21^WAF1/CIP1^ and p27^KIP1^ for degradation, Tax stabilizes these two CKIs. On the contrary, the association of chicken anemia virus protein Apoptin with APC1 inhibits the activity of theAPC/C ubiquitin ligase, leading to the stabilization of cyclin B1 and other APC/C substrates, with ensuing cell cycle G2/M arrest and apoptosis (Teodoro et al., 2004).

The deubiquitinase (DUB) USP37 has been found to stabilize cyclin A by removing the polyubiquitin from the latter and, hence, accelerates entry into S phase (Huang et al., 2011). HBx of HBV could upregulate USP37 transcripts in both hepatic and non-hepatic cells and also prevents USP37 from proteasomal degradation. HBx acts as a chaperone of USP37 and shuttles it out of the nucleus, where the ubiquitin E3 ligase CDC20 homolog 1 (CDH1) and b-TrCP associate with USP37 (Zhou et al., 2003; von Mikecz, 2006; Saxena and Kumar, 2014). Thus, by stabilizing the expression of USP37, HBx promotes the accumulation of cyclin A to modulate cell cycle progression. Furthermore, HBx hinders the proteolytic cleavage of Notch1, which plays a critical role in proliferation (Artavanis-Tsakonas et al., 1999; Wang et al., 2010; Xu et al., 2010). This is achieved through HBx-mediated transcriptional downregulation of presenilin 1, a catalytic subunit of the large protease complex γ-secretase which mediates the cleavage of Notch 1 (Artavanis-Tsakonas et al., 1999; Kopan and Ilagan, 2009; Xu et al., 2010).

In addition to hijacking the host ubiquitination machinery, viruses could also encode their own degradation enzymes. The zinc-binding RING finger domain of ICP0 of HSV-1 possesses a ubiquitin E3 ligase activity (Boutell et al., 2002; Hagglund et al., 2002). Sole expression of ICP0 is able to induce rapid loss of the centromeric protein CENP-C and CENP-A in a proteasome-dependent manner, resulting in mitotic block (Everett et al., 1999; Lomonte et al., 2001). However, it remains unclear whether these two centromere components are direct substrates of the ICP0 E3 ligase. The use of purified p53 in the presence of ICP0, together with a full complement of the ubiquitin-conjugating enzymes in test tubes, revealed a direct ubiquitination of p53 by ICP0 (Boutell and Everett, 2003); p53 levels, howver, were not reduced during HSV-1 infection and, hence, the role of p53 in ICP0-induced perturbation of cell cycle progression and accumulation in G1/S and G2/M needs further investigation (Hobbs and DeLuca, 1999). Still, it provides a novel mechanism by which a virus-encoded protein functions as an E3 ligase to catalyze the degradation of cell cycle checkpoint proteins.

Acute Japanese encephalitis virus (JEV) infection leads to cell cycle G0/G1 arrest with elevated p21^WAF1/CIP1^ and p27^KIP1^ levels, and reduced production of cyclin D (Das and Basu, 2008). However, in the case of persistent JEV infection, expression of these CKIs and GSK-3β, which mediates the degradation of cyclin D, is suppressed (Diehl et al., 1998; Kim et al., 2015). Thus, by extending the stability of cyclin D, persistent JEV infection bypasses cell cycle arrest, striking a balance between the persistence of lytic RNA virus and host survival. Enterovirus 71 (EV71), the etiological agent of hand, foot, and mouth disease, was shown to prevent cell cycle transition from S to G2/M phase (Ooi et al., 2007; Yu et al., 2015). Although the molecular mechanisms is still unclear, it was observed that cyclin A2 was targeted for proteasome-mediated degradation during infection (Yu et al., 2015).

## Subversion by protein redistribution (Table 4 and Figure 4)

While it is well established that several biochemical processes serve to orchestrate cell cycle control, including protein-protein interaction and dissociation, phosphorylation/ dephosphorylation, and the synthesis/ destruction of cell cycle regulators at specific time point, the importance of subcellular localization of CDK-cyclins complexes and their regulators is receiving increasing attention for proper cell-cycle coordination. Hence, viruses have also evolved mechanisms to deregulate cell cycle progression by targeting the localization of cell cycle regulators.

**Table 4:** Cell cycle subversion by protein redistribution.

**Virus/Genome**	**Viral protein**	**Host protein**	**Functional consequence**	**References**
HIV-1/RNA plus	Vpr	14-3-3σ scaffolding protein, MAPKAP kinase-2	Sequestration of Cdc25C in cytoplasm, G2/M arrest	Kino et al., 2005; Bolton et al., 2008; Huard et al., 2008
HIV-1/RNA plus	Vif	CDK1, cyclin B1	Cytoplasmic retention, G2/M arrest	Sakai et al., 2011
HHV-6-HHV8/dsDNA		E2F1	Accumulation in cytoplasm, reduction of E2F1-Rb complexes and cell cycle arrest	Mlechkovich and Frenkel, 2007; Sharon et al., 2014
Influenza A/RNA minus	M2	Cyclin D3	Redistribution of cyclin D3 from the nucleus to the cytoplasm; cell cycle arrest in G0/G1	Fan et al., 2017
HCMV/dsDNA		CDK2	Nuclear translocation, mitogenic	Bresnahan et al., 1997
HCMV/dsDNA	NS5A	p53	Reduced nuclear translocation, ant-apoptotic	Kovacs et al., 1996; Fortunato and Spector, 1998; Majumder et al., 2001
Parvovirus B19/ssDNA	NS1	E2F4-8 repressive transcription factors	Nuclear import and G2 arrest	Wan et al., 2010
Epstein-Barr/dsDNA	LMP1	Survivin, p53	Increased nuclear localization	Guo et al., 2012
Avian Reovirus/dsRNA	P17	CDK1	Cytoplasmic retention	Chiu et al., 2018

**Figure 4:**
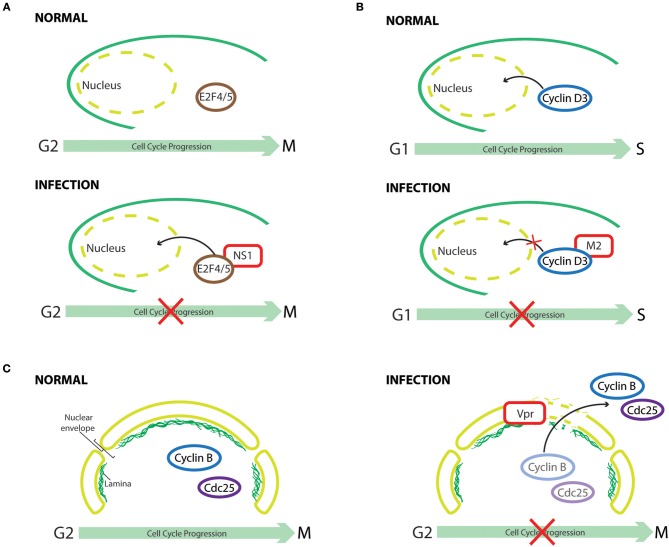
Cell cycle subversion by protein redistribution. **(A)** Nonstructural protein 1 (NS1) of human parvovirus B19 (B19V) leads to G2 arrest, which is mediated by upregulating nuclear localization of repressive E2F4 and E2F5 transcription factors (Wan et al., 2010). **(B)** Cyclin D3 needs to be imported into the nucleus to assist G0/G1 cell cycle progression (Mahony et al., 1998). Influenza A virus infection perturbs cell cycle progression by redistributing cyclin D3 from the nucleus to the cytoplasm, triggering its proteasomal degradation (Fan et al., 2017). **(C)** Expression of HIV-1 Vpr induces localized-disruption in the lamin architecture, resulting in G2/M arrest that is dependent on cytoplasmic compartmentalization of Cdc25C, cyclin B1, and Wee1 (Peng et al., 1997; Laronga et al., 2000; de Noronha et al., 2001; Kino et al., 2005).

One early example of virus-induced translocation of cell cycle regulators is exemplified by HCMV infection, which can mitogenically stimulate growth-arrested cells to enter the cell cycle by promoting nuclear uptake of CDK2 (Bresnahan et al., 1997). This suggests that HCMV possesses mechanisms to regulate the subcellular distribution of CDK2, thus facilitating the activation of cyclin E-CDK2 complex and, consequently, modulating cell cycle progression. It is possible that CDK2 could be transported into the nucleus in a “piggyback” manner, interacting with a viral protein that shuttles between nucleus and cytoplasm, or that viral infection activates the intrinsic CDK2 import machinery. Indeed, infection of primary erythroid progenitor cells by human parvovirus B19 (B19V) leads to G2 arrest, which is dependent on viral nonstructural protein 1 (NS1), as its sole expression could recapitulate the same phenomenon (Wan et al., 2010). The cell cycle perturbation effect results from NS1-mediated downregulation of activating E2Fs transcription factors (E2F1 to E2F3a) and upregulation of repressive E2Fs (E2F4 to E2F8). Interestingly, co-immunoprecipitation experiments in NS1 expressing cells identified NS1-E2F4 and NS1-E2F5 complexes, which could enhance the nuclear import of these transcription-repressive factors. Normal cell cycle progression was observed in cells infected with a mutant B19V, which encodes a nuclear-localization-deficient NS1, demonstrating the importance of NS1-mediated redistribution of E2F4 and E2F5 in causing cell cycle perturbation (Wan et al., 2010).

For cyclin D3 to exert its effect in cell cycle G0/G1 progression, it needs to be transported from the cytoplasm to the nucleus (Mahony et al., 1998). It was demonstrated through confocoal imaging and subcellular fractionation assay that, in influenza A virus infected cells, cyclin D3 was significantly redistributed from the nucleus to the cytoplasm, followed by its proteasomal degradation (Fan et al., 2017). Hence, through multiple mechanisms, influenza A virus perturbs cell cycle progression for enhanced progeny virus production. Similarly, a significant increase in the cytoplasmic level of CDK1 was observed in ARV-infected or p17-expressing cells (Chiu et al., 2018). Thus, p17 of ARV not only perturbs cell cycle progression into mitosis through direct inhibition of CDK1 kinase activity, but also disrupts CDK1/cyclin B1 complex formation in the nucleus by sequestering CDK1 in the cytoplasm. Although total p53 levels were elevated after infection, a reduction of p53 level in nuclear extracts of HCMV-infected human umbilical vein endothelial cells was observed, resulting in an anti-apoptotic phenotype of HCMV-infected cells (Kovacs et al., 1996). The reduced nuclear-translocation of p53 by HCMV, as suggested by immunofluoresence, provides a plausible explanation for the oncogenic potential of HCMV (Kovacs et al., 1996). Wang et al. later reported that the cytoplasmic sequestration of p53 by HCMV occurs by blocking the nuclear localization signal (NLS) of p53 (Wang et al., 2001). Interestingly, in addition to perturbing the nuclear-import of p53, HCMV could also inhibit the function of nuclear p53 through sequestering it into discrete foci, viz., the replication centers, within the nuclei of HCMV-infected cells (Fortunato and Spector, 1998). Changes in the subcellular localization of p53 were also observed in two different cell lines that expressed the HCV NS5A protein (Majumder et al., 2001). Endogenous p53 is a nuclear protein, whereas it was retained at the perinuclear membrane following NS5A expression (Shaulsky et al., 1991). A physical association of NS5A with p53 was demonstrated both *in vitro* and *in vivo*; however, it is not the cause for this sequestration as NS5A binding-deficient p53 was still observed to localize on the perinuclear membrane. In accordance, hTAF_II_32, a transcriptional coactivator of p53, was also partially sequestered by NS5A in the cytoplasm, thus suggesting an indirect mechanism (Lu and Levine, 1995; Lan et al., 2002). Although p53 is known as a tumor suppressor gene, increasing evidence suggest that nasopharyngeal carcinoma (NPC) is associated with overexpression and accumulation of p53 (Gulley et al., 1998; Yip et al., 2006). It is interesting to find that the late membrane protein 1 (LMP1) of EBV not only upregulates the expression and phosphorylation of p53 and survivin, which possesses a p53-binding element in its promoter region and facilitates G1/S progression by interacting with CDK4 in the nucleus (Suzuki et al., 2000; Hoffman et al., 2002; Mirza et al., 2002; Guo et al., 2012), but also increases the nuclear localization of both proteins (Guo et al., 2012).

Expression of HIV-1 Vpr could induce localized-disruption in the lamin architecture, which is known to interfere with DNA synthesis and changes in the intracellular trafficking of cell cycle regulators (Moir et al., 2000; de Noronha et al., 2001). This structural defect of lamin leads to the formation of nuclear envelope herniations as revealed by immunofluorescence, resulting in changes in the subcellular compartmentalization of Cdc25C, cyclin B1, and Wee1 (de Noronha et al., 2001). Thus, interaction of HIV-1 Vpr with the 14-3-3σ scaffolding protein, a G2/M transition regulator that inactivates Cdc25C by changing its subcellular localization and/or stability (Fu et al., 2000; Muslin and Xing, 2000), has been implicated in Vpr-induced cell cycle perturbation (Peng et al., 1997; Lopez-Girona et al., 1999; Laronga et al., 2000; Kino et al., 2005). Vpr could mediate protein assembly among 14-3-3σ, Cdc25C, CDK1, and cyclin B1, leading to G2/M arrest by either sequestering Cdc25 in the cytoplasm or impairing the normal association of 14-3-3σ with centrosomal proteins (Kino et al., 2005; Bolton et al., 2008). An association of Vpr with MAPKAP kinase-2 (MK2), a possible regulatory kinase of Cdc25, was reported by Huard et al. (2008). Although the relevance of this interaction in Vpr-mediated cell cycle arrest needs further investigation, it appears that MK2 is required for Vpr-induced Cdc25 phosphorylation and nuclear export (López-Avilés et al., 2005; Manke et al., 2005; Huard et al., 2008). The accessory protein viral infectivity factor (Vif) of HIV-1 also inhibits cell cycle progression at the G2 phase by interfering with the nuclear translocation of CDK1 and cyclin B1, a finding corroborated by infection experiments with Vif-deficient HIV-1 virus that point to a role of this viral protein in mediating CDK1 cytoplasmic-retention (Sakai et al., 2011).

HHV-6A and HHV-6B provide other examples of mislocalization coupled to cell cycle arrest, as their infection reduces E2F1-Rb complex formation and leads to accumulation of E2F1 both in the cytoplasmic and nuclear fractions, as opposed to its strict nuclear localization in normal cells (Mlechkovich and Frenkel, 2007). Although the relevance of E2F1 translocation in HHV-6-induced cell cycle arrest is not completely understood, it illustrates that viruses could also target E2F family of protein in this context (Sharon et al., 2014).

## Subversion by virus-encoded homologs of cell cycle regulators (Table 5 and Figure 5)

Herpesvirus saimiri (HVS), a T-lymphotropic gamma-herpesvirus that establishes persistent conditions in primate host species, encodes the first reported viral-cyclin (v-cyclin) (Nicholas et al., 1992; Fickenscher and Fleckenstein, 2001). Similarly, amino acid and DNA sequence analyses of KSHV, which has been strongly implicated as the etiologic factor in the development of Kaposi's sarcoma and primary effusion lymphomas (PEL) (Chang et al., 1994; Cesarman et al., 1995), identified a “cyclin-box” showing similarity to human cyclin D2 (Chang et al., 1996; Li et al., 1997). In addition, murine gammaherpesvirus 68 (MHV68), another member of the gammaherpesviruses, shares with HVS and KSHV similarities in the sequence of an open reading frame that is predicted to encode a D-type cyclin (Virgin et al., 1997). Taken together, these findings argue for a conserved role of these v-cyclins in gammaherpesvirus biology. Indeed, both HVS and KSHV v-cyclins show strong association with CDK6, leading to the phosphorylation of Rb and histone H1 (Jung et al., 1994; Li et al., 1997). Surprisingly, despite the fact that v-cyclin of MHV68 displays only around 20% similarity to cyclin A/E, it associates with CDK2 rather than CDK6 (Card et al., 2000). Herpesvirus ateles (AtHV-2) causes lethal lymphomas in various new world primates and is closely related to HVS. The light DNA segment of AtHV-2 possesses an open reading frame which shows significant homology to D-type cyclins although its function was not studied in molecular terms (Albrecht, 2000).

**Table 5:** Cell cycle subversion by virus-encoded homologs of cell cycle regulators.

**Virus/Genome**	**Viral protein**	**Host protein**	**Functional consequence**	**References**
Herpesvirus saimiri, KSHV/dsDNA	Viral cyclins	CDK6	Rb and H1 phosphorylation; CAK-independent activation of CDK6	Jung et al., 1994; Godden-Kent et al., 1997; Li et al., 1997; Swanton et al., 1997; Kaldis, 1999
MHV68/dsDNA	Viral cyclin	CDK2	Phosphorylation of p27^KIP1^ to bypass G1 arrest	Virgin et al., 1997; Card et al., 2000
KSHV/dsDNA	Viral cyclin	p27^KIP1^	Phosphorylation-dependent degradation, cell cycle progression	Vlach et al., 1997; Mann et al., 1999; Zhi et al., 2015
*Autographa californica* nucleopolyhedrovirus /dsDNA	IE2, occlusion-derived viral form-EC27	CDK6, CDK1	Rb phosphorylation, G2/M cell cycle arrest	Belyavskyi et al., 1998; Braunagel et al., 1998; Prikhod'ko and Miller, 1998
Walleye dermal sarcoma virus/RNA plus	Retroviral cyclins	CDK8, CDK3	Cell proliferation	LaPierre et al., 1998; Brewster et al., 2011
Poxviruses/dsDNA	Poxvirus APC/cyclosome regulator	APC2 scaffold protein	Impairment of APC/C function, accumulation of cells in G2/M	Mo et al., 2009

**Figure 5:**
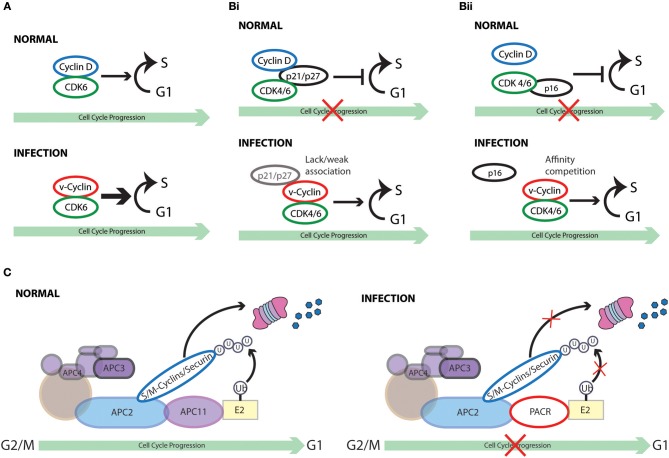
Cell cycle subversion by virus-encoded homologs of cell cycle regulators. **(A)** Several gammaherpesviruses encode viral-cyclins (v-cyclin) (Nicholas et al., 1992; Chang et al., 1994, 1996; Cesarman et al., 1995; Li et al., 1997; Virgin et al., 1997; Fickenscher and Fleckenstein, 2001) that display strong association with CDK6, leading to the phosphorylation of Rb and histone H1. **(Bi)** V-cyclin-CDK complexes are significantly more resistant to the inhibition imposed by p21^WAF1/CIP1^, p27^KIP1^, and p16^INK4A^ than cyclin D1-CDK complexes *in vitro* (Swanton et al., 1997). Failure or weak association of p21^WAF1/CIP1^ and p27^KIP1^ with v-cyclin likely defines the inefficiency of these CKI to inhibit them (Swanton et al., 1997; Schulze-Gahmen et al., 1999; Card et al., 2000). **(Bii)** Higher affinity of v-cyclin for CDK6 than p16^INK4A^ may prevent its displacement by p16^INK4A^. **(C)** Both crocodile and squirrel poxviruses encode the APC/cyclosome regulator (PACR), which shares sequence similarities to the APC subunit 11 (APC11). PACR mimics the binding ability of APC11 to APC2 but not the ubiquitin ligase activity of APC11. Thus, poxvirus hinders normal cell cycle progression by encoding an inactive homolog of APC11 (Mo et al., 2009).

Interestingly, unlike D-type cyclins that have broad association with different CDKs, the v-cyclins of HVS and KSHV bind almost exclusively to CDK6 (Godden-Kent et al., 1997). Of note, KSHV-cyclin can activate CDK6 independent of phosphorylation by CAK *in vitro* and *in vivo* (Kaldis, 1999). Moreover, the kinase activity of v-cyclin-CDK6 appears to be much stronger than cyclin D1-CDK6, targeting also histone H1, which is a preferred substrate of CDK2 rather than CDK6 (Alexandrow and Hamlin, 2005). This finding suggests a transformation potential of v-cyclin through “hyperactivation” of CDK6 (Jung et al., 1994; Swanton et al., 1997). In fact, ectopic expression of KSHV v-cyclin abolishes the cell cycle arrest effect imposed by Rb, which is related to Rb hyperphosphorylation and functional inactivation (Chang et al., 1996). In support of these finding, MHV v-cyclin-transgenic mice display a significant increase in thymocyte number with promoted cell cycle progression and also develop high-grade lymphoblastic lymphoma (van Dyk et al., 1999).

Furthermore, the v-cyclin-CDK complexes are significantly more resistant to the inhibition imposed by p21^WAF1/CIP1^, p27^KIP1^, and p16^INK4A^ than cyclin D1-CDK complexes *in vitro* (Swanton et al., 1997). Lack or weak association of p21^WAF1/CIP1^ and p27^KIP1^ with v-cyclin likely defines the inefficiency of these CKI to inhibit them (Swanton et al., 1997; Schulze-Gahmen et al., 1999; Card et al., 2000). It is currently unclear how v-cyclins antagonize the inhibitory effect of p16^INK4A^ since this CKI acts by inhibiting the catalytic subunit of CDK4 and CDK6 (Sherr and Roberts, 1999). One hypothesis is that higher affinity of v-cyclin for CDK6 than p16^INK4A^ may prevent its displacement by p16^INK4A^. Deletion of the p16^INK4A^ locus was identified in KSHV associated PEL primary samples, suggesting that loss of p16^INK4A^ may be another mechanism of KSHV-induced transformation (Platt et al., 2002). In addition, by targeting p27^KIP1^ to phosphorylation-dependent degradation, the v-cyclin of KSHV could prevent the G1 arrest induced by the viral latency protein vFLIP, which is co-translated from the same viral mRNA as v-cyclin (Vlach et al., 1997; Mann et al., 1999; Zhi et al., 2015). This was also demonstrated by the inability of KSHV v-cyclin to escape p27^KIP1^ mediated cell cycle arrest when p27^KIP1^ is mutated to a form that cannot be phosphorylated. Moreover, p27^KIP1^ phosphorylation on Ser10 by KSHV v-cyclin-CDK6 complex enhances its cytoplasmic dislocation, which serves as another means to facilitate cell cycle progression (Boehm et al., 2002; Ishida et al., 2002; Sarek et al., 2006). Similarly, CDK2 associated with v-cyclin of MHV68 exhibits substantial resistance to both p21^WAF1/CIP1^ and p27^KIP1^, while inducing phosphorylation of p27^KIP1^ to overcome p27^KIP1^-mediated G1 arrest (Yarmishyn et al., 2008). However, v-cyclin of MHV68 is only able to subvert p27^KIP1^-imposed G1 arrest, but not that imposed by p21^WAF1/CIP1^ (Yarmishyn et al., 2008).

*Autographa californica* nucleopolyhedrovirus (AcMNPV) infects pest insect and causes cycle arrest at G2/M phase in host cells with continuing viral DNA replication (Braunagel et al., 1998). Sole expression of the IE2 protein of AcMNPV results in accumulation of enlarged cells with DNA content greater than 4N (Prikhod'ko and Miller, 1998). More interestingly, the structural protein occlusion-derived viral form (ODV)-EC27 is endowed with cyclin-like activity, making it the second viral-encoded cyclin homolog reported (Belyavskyi et al., 1998). Similar to the v-cyclins of herpesviruses, association of ODV-EC27 with CDK6 leads to Rb phosphorylation (Huard et al., 2008). Additionally, ODV-EC27 was found to interact with CDK1, exerting cyclin B-like activity.

Walleye dermal sarcoma virus (WDSV) and walleye epidermal hyperplasia virus (WEHV) are retroviruses that encode human D-type cyclin homologs. A standard yeast complementation assay showed that the retroviral-cyclins (rv-cyclins) of WDSV, but not that of WEHV, induce cell cycle progression in G1-cyclin-deficient yeast (LaPierre et al., 1998). In addition to sharing sequence similarity with D-type cyclins, WDSV rv-cyclin was shown to be a structural ortholog of cyclin C (Brewster et al., 2011), which was identified owing to its ability to rescue cyclin G1 function in yeast (Huard et al., 2008). Cyclin C has been shown to promote G0/G1 transition through phosphorylating Rb in combination with CDK3 (Lew et al., 1991; Ren and Rollins, 2004) and was later recognized as part of the Mediator complex, which links gene-specific activators to the general RNA Pol II transcription machinery, in complex with CDK8 (Tassan et al., 1995; Conaway and Conaway, 2011; Malumbres, 2014). As expected, WDSV rv-cyclin has been shown to interact with either CDK8, component of the Mediator complex (Rovnak and Quackenbush, 2002; Poss et al., 2013), or CDK3, leading to enhanced host gene expression or cell proliferation without serum stimulation, respectively (Brewster et al., 2011). Other viruses have been shown to interact with subunits of the Mediator complex, but they are doing so independently of viral cyclins. For example, varicella-zoster virus (VZV), an alpha-herpes virus that causes varicella and zoster, targets the MED25 subunit of Mediator through its major transactivator protein, immediate early gene 62 (IE62) (Yang et al., 2008), whereas herpes simplex virus utilizes VP16, which controls the transcription of immediate early genes, to activate transcription through a specific MED25-associated mediator deficient in CDK8 (Uhlmann et al., 2007). Interestingly, it was later found that CDK8 could positively regulate VP16-dependent transcriptional regulation, while CDK11 and CDK19 exhibit opposing roles (Furumoto et al., 2007; Tsutsui et al., 2008, 2011).

Sequence analysis of available poxvirus genome has revealed that both crocodile and squirrel poxviruses encode a RING-H2 protein, the poxvirus APC/cyclosome regulator (PACR),which shares sequence similarities to the APC subunit 11 (APC11). Together with the scaffold protein APC2, APC11 forms the catalytic core of APC/C, which regulates the progression through mitosis by targeting several anaphase inhibitory proteins for proteolysis and the maintenance of G1 phase (Tang et al., 2001; Hsu et al., 2002; Song et al., 2004; Wei et al., 2004; Wehman et al., 2007; Mo et al., 2009). Interestingly, PACR mimics the binding ability of APC11 to APC2 but not the ubiquitin ligase activity of APC11. Expression of PACR impairs APC/C function, accompanied by the accumulation of cells in G2/M phase. The cell cycle perturbation effect of PACR requires its N-terminal region, which is also the region responsible for association with APC2 (Mo et al., 2009). Thus, poxvirus hinders normal cell cycle progression by encoding an inactive homolog of APC11.

## Subversion by other mechanisms (Table 6)

HIV-1 Vpr-induced G2 arrest depends on the activation of ATR, the ataxia telangiectasia mutated (ATM)- and Rad3-related protein, which phosphorylates Chk1 and, in turn, leads to both phosphorylation-induced degradation of Cdc25A and Cdc25C cytosolic sequestration (Peng et al., 1997; Sanchez et al., 1997; Zhao and Piwnica-Worms, 2001; Zhao et al., 2002; Roshal et al., 2003). It has been proposed that the activation of ATR-dependent DNA damage G2/M checkpoint by Vpr involves the direct binding of this viral protein to chromatin, as mutants which are unable to bind chromatin fail to activate ATR (Lai et al., 2005), although the underlying mechanism remains controversial. Lai et al. reported that Vpr does not induce DNA double-strand breaks (DSBs), but increases the association of replication protein A (RPA) with chromatin, an upstream event required for ATR activation (Cortez et al., 2001; Zou and Elledge, 2003; Lai et al., 2005). However, using the same pulsed-field gel electrophoresis assay, Tachiwana et al. later showed that Vpr expression could induce chromosomal DSBs (Tachiwana et al., 2006). Differences in experimental settings may underpin this discrepancy. Lai et al. relied on Vpr-transfected cells for the PFGE analysis, whereas isolated nuclei that were treated with purified Vpr were used in the other study. The NS1 of the autonomous parvovirus minute virus of mice (MVMp) is another example of viral protein that affects the integrity of chromatin. MVMp infection leads to cell cycle arrest in S phase as a result of NS1-induced nicks in the cellular chromatin (Op De Beeck et al., 1995; Op De Beeck and Caillet-Fauquet, 1997).

**Table 6:** Cell cycle subversion by other mechanisms.

**Virus/Genome**	**Viral protein**	**Host protein**	**Functional consequence**	**References**
Parvovirus minute virus of mice /ssDNA	NS1	Chromatin	Nicks affecting integrity, cell cycle arrest in S phase	Op De Beeck et al., 1995; Op De Beeck and Caillet-Fauquet, 1997
HIV-1/RNA plus	Vpr	Chromatin	Activation of DNA damage response, G2 arrest	Roshal et al., 2003; Lai et al., 2005
KSHV/dsDNA	miRNA K1	p21^WAF1/CIP1^	Inhibition of p21^WAF1/CIP1^ expression, cell cycle progression	Gottwein and Cullen, 2010
Adeno-associated virus/ssDNA	Viral genome	p53, p21^WAF1/CIP1^	Activation of DNA damage response, cell cycle arrest	Raj et al., 2001
*Bocavirus* minute virus of canines/ssDNA	Viral genome		G2/M arrest	Sun et al., 2009; Chen et al., 2010
Measles virus/RNA minus	Hemagglutinin, fusion protein		Inhibition of T-cell expansion, accumulation in G0/G1	Dubois et al., 2001; Griffin, 2010
Epstein-Barr/dsDNA	miRNAs (BHRF1 locus)		Inhibition of apoptosis and cell cycle progression	Seto et al., 2010
Zika virus/RNA plus	M, E, NS4A	p53, p21^WAF1/CIP1^	G2/M cell cycle arrest	Li et al., 2017; Liu et al., 2018
Zika virus/RNA plus	prM		G1 accumulation	Li et al., 2017
Avian Reovirus/dsRNA	p17	Tpr nucleoporin	Nuclear accumulation and activation of p53, p21^WAF1/CIP1^ and PTEN, cell cycle arrest	Huang et al., 2015

Infection of adeno-associated virus (AAV) leads to accumulation of cells with 4N DNA content in p53-competent cells, whereas it induces apoptosis in p53-deficient cells (Raj et al., 2001). Interestingly, virus replication or viral protein expression is not required for AAV-mediated cell cycle arrest as ultraviolet-treated AAV maintain their ability to perturb cell cycle, pointing to the role of AAV genome in these effects (Raj et al., 2001). In support of this hypothesis, empty AAV particles, which lack viral genome, fail to affect cell growth (Raj et al., 2001). It was proposed that the special structure of AAV genome, a single-stranded DNA with hairpin loops at both ends, could trigger host DNA damage response and mediate cell cycle arrest in a p53/p21^WAF1/CIP1^-dependent manner (Raj et al., 2001). Indeed, using an oligonucleotide corresponding to the AAV hairpin structure but not the AAV coding sequence, the same group successfully replicated the phenotypes induced by AAV infection (Raj et al., 2001). Likewise, expression of the individual proteins of *Bocavirus* minute virus of canines (MVC) has no effect on cell cycle progression (Chen et al., 2010). However, inoculation of UV-inactivated virus or a nonreplicative mutant virus phenocopied the G2/M arrest induced by MVC infection, suggesting that viral genome counts for the effect (Chen et al., 2010). Indeed, the use of a series of MVC mutant constructs indicated the terminal repeats of its genome, which form a strong secondary structure, are essential in the G2/M arrest feature of MVC (Sun et al., 2009; Chen et al., 2010).

Other than viral proteins and genome, virus-encoded microRNAs (miRNAs) have been also implicated to play a role in regulating host cell cycle. For example, KSHV miRNA K1 specifically represses the expression of p21^WAF1/CIP1^ via the 3′-UTR of this CKI, antagonizing p21^WAF1/CIP1^-mediated cell cycle arrest (Gottwein and Cullen, 2010). The significance of viral miRNA in favoring cell cycle progression was studied by introducing mutations into the precursor miRNA transcripts of EBV, which prevents the subsequent production of mature viral miRNAs thus antagonizes both early B-cell proliferation and miRNA protection from spontaneous apoptosis (Seto et al., 2010).

Measles virus (MV) was reported to subvert dendritic cells (DCs) and induce immune suppression. Dubois et al. found that infection of DCs by MV led to their functional maturation, allowing the migration of DCs from mucosal surfaces to draining lymph nodes to prime naive T cells (Dubois et al., 2001). Interestingly, instead of activating T cells, MV-infected DCs block T-cell expansion through cell-to-cell contact. The use of anti-MV HA or fusion protein (F) antibodies revealed that these two viral proteins, which are expressed on virus-infected DCs, are responsible for the suppression of T-cell proliferation (Dubois et al., 2001). Thus, through surface expression of HA and F on virus-infected DCs, MV takes a free ride on DCs to reach draining lymph nodes and subverts the normal function of DCs to inhibit T-cell proliferation. This infection-independent action in T cells results in accumulation of cells in G0/G1, owing to the inhibition of PI3K signaling in response to T-cell receptor binding (Griffin, 2010).

Zika virus (ZIKV), a flavivirus that is transmitted to humans primarily by *Aedes* mosquitoes, has been shown to cause congenital microcephaly when it infects women early in pregnancy (de Araújo et al., 2018; Hoen et al., 2018). This has led to a flurry of studies in different experimental models that have established the ZIKV induces massive abnormalities of cell division, including the presence of supernumerary centrosomes, that lead to virally-induced caspase 3-mediated apoptotic cell death and cell cycle abnormalities of human neural progenitor cells (Faizan et al., 2016; Souza et al., 2016; Gabriel et al., 2017), although how it interferes with the cell cycle has not been precisely defined. Moreover, transcriptional profiling of infected human neural progenitor cells has revealed a dysregulation of cell cycle- and differentiation-related pathways in neural progenitor cells (Dang et al., 2016; Li et al., 2016; Tang et al., 2016). Interestingly, it has been observed that an antiproliferactive and pro-apoptotic p53-related response markedly different from p53-induced transcriptional changes upon infection by HCMV, which can also cause microcephaly, is activated in ZIKA-infected human neural progenitor cells (Ghouzzi et al., 2016). More recently, by investigating the effect of individual ZIKV proteins *in vitro*, it was found that expression of the membrane-anchored capsid anaC, structural envelope E, membrane M proteins, and nonstructural protein 4A (NS4A) could trigger G2/M cell cycle arrest and apoptosis (Li et al., 2017; Liu et al., 2018). The NS4A induces growth restriction via the target of rapamycin (TOR) cellular stress pathway (Li et al., 2017), whereas envelope-triggered apoptosis is associated with upregulation of both p53 and p21^Cip1^*/*^Waf1^ and downregulation of cyclin B1 (Liu et al., 2018). Interestingly, ZIKA membrane protein (M) induce arrest of the cell cycle in G2/M, whereas the pre-membrane protein (prM) results in G1 accumulation (Li et al., 2017), revealing that posttranslational processing modifies its interference with cell cycle progression.

## Conclusions

The interplay between the life cycle of viruses and the series of events leading to cell division has been actively investigated since the discovery that certain tumors were associated to viral infection. Although estimates of the incidence of cancers caused by viruses vary, both DNA and RNA viruses have been clearly linked to the development of human cancers (Liao, 2006; Mesri et al., 2014). However, most viruses have evolved a more complicated relationship with the cell cycle contenting themselves with surreptitious tampering for more effective replication and productive infection, without leading to cell transformation and tumorigenesis. The most obvious mechanism is the direct interaction of a viral gene product with a host protein to disarm its function, which has been adopted by many viruses as a common strategy to exploit host cell cycle. It is commonly held that, by interacting with cell cycle regulators and delaying cell division, these viruses can subvert the biosynthetic apparatus, re-directing it from the production of host proteins needed for cell cycle progression to suit their replication needs. Viruses stop at nothing and use any means necessary to survive in a hostile environment by inducing posttranslational modifications and dislocating host restriction factors to modulate their function and/or degradation. Co-evolution and adaptation of virus-host interactions is best described as a zero-sum biological arms race. It is clear that viruses could utilize different nodes of the cell cycle machinery to hamper cell division to escape the dependency on the normal progression of cellular proliferation. The mechanistic study of these interactions has revealed unexpected, non-canonical roles of cellular proteins. Further work should bring about a more refined understanding that will shed new light on the panoply of assets available to viruses to subvert the process of cell division, while at the same time providing insight into the function of host proteins that can offer unexpected handles to counteract viral infection.

## Author contributions

All authors listed have made a substantial, direct and intellectual contribution to the work, and approved it for publication.

### Conflict of interest statement

The authors declare that the research was conducted in the absence of any commercial or financial relationships that could be construed as a potential conflict of interest.

## References

[B1] Ahn J. Y. Schwarz J. K. Piwnica-Worms H. Canman C. E. (2000). Threonine 68 phosphorylation by ataxia telangiectasia mutated is required for efficient activation of Chk2 in response to ionizing radiation. Cancer Res 60, 5934–5936. 11085506

[B2] Albrecht J. C. (2000). Primary structure of the Herpesvirus ateles genome. J. Virol. 74, 1033–1037. 10.1128/JVI.74.2.1033-1037.2000 10623770 PMC111628

[B3] Aleem E. Kiyokawa H. Kaldis P. (2005). Cdc2-cyclin E complexes regulate the G1/S phase transition. Nat. Cell Biol. 7, 831–893. 10.1038/ncb1284 16007079

[B4] Alexandrow M. G. Hamlin J. L. (2005). Chromatin decondensation in S-phase involves recruitment of Cdk2 by Cdc45 and histone H1 phosphorylation. J. Cell Biol. 168, 875–886. 10.1083/jcb.200409055 15753125 PMC2171796

[B5] Ando T. Kawabe T. Ohara H. Ducommun B. Itoh M. Okamoto T. (2001). Involvement of the interaction between p21 and proliferating cell nuclear antigen for the maintenance of G2/M arrest after DNA damage. J. Biol. Chem. 276, 42971–42977. 10.1074/jbc.M106460200 11559705

[B6] Artavanis-Tsakonas S. Rand M. D. Lake R. J. (1999). Notch signaling: cell fate control and signal integration in development. Science 284, 770–776. 10.1126/science.284.5415.770 10221902

[B7] Baer A. Austin D. Narayanan A. Popova T. Kainulainen M. Bailey C.. (2012). Induction of DNA damage signaling upon Rift Valley fever virus infection results in cell cycle arrest and increased viral replication. J. Biol. Chem. 287, 7399–7410. 10.1074/jbc.M111.296608 22223653 PMC3293538

[B8] Bagchi S. Raychaudhuri P. Nevins J. R. (1990). Adenovirus E1A proteins can dissociate heteromeric complexes involving the E2F transcription factor: a novel mechanism for E1A trans-activation. Cell 62, 659–669. 10.1016/0092-8674(90)90112-R 2143697

[B9] Baldin V. Lucas J. Marcote M. J. Pagano M. Draetta G. (1993). Cyclin D1 is a nuclear protein required for cell cycle progression in G1. Genes Dev. 7, 812–821. 10.1101/gad.7.5.812 8491378

[B10] Banerjee N. S. Wang H. K. Broker T. R. Chow L. T. (2011). Human papillomavirus (HPV) E7 induces prolonged G2 following S phase reentry in differentiated human keratinocytes. J. Biol. Chem. 286, 15473–15482. 10.1074/jbc.M110.197574 21321122 PMC3083224

[B11] Barbosa M. S. Edmonds C. Fisher C. Schiller J. T. Lowy D. R. Vousden K. H. (1990). The region of the HPV E7 oncoprotein homologous to adenovirus E1a and Sv40 large T antigen contains separate domains for Rb binding and casein kinase II phosphorylation. EMBO J. 9, 153–160. 10.1002/j.1460-2075.1990.tb08091.x 2153075 PMC551641

[B12] Bartek J. Bartkova J. Lukas J. (1996). The retinoblastoma protein pathway and the restriction point. Curr. Opin. Cell Biol. 8, 805–814. 10.1016/S0955-0674(96)80081-0 8939678

[B13] Bartek J. Lukas J. (2003). Chk1 and Chk2 kinases in checkpoint control and cancer. Cancer Cell 3, 421–429. 10.1016/S1535-6108(03)00110-7 12781359

[B14] Baydoun H. H. Pancewicz J. Bai X. Nicot C. (2010). HTLV-I p30 inhibits multiple S phase entry checkpoints, decreases cyclin E-CDK2 interactions and delays cell cycle progression. Mol. Cancer 9:302. 10.1186/1476-4598-9-302 21092281 PMC3000403

[B15] Belyavskyi M. Braunagel S. C. Summers M. D. (1998). The structural protein ODV-EC27 of Autographa californica nucleopolyhedrovirus is a multifunctional viral cyclin. Proc. Natl. Acad. Sci. U.S.A. 95, 11205–11210. 10.1073/pnas.95.19.11205 9736714 PMC21620

[B16] Benn J. Schneider R. J. (1995). Hepatitis B virus HBx protein deregulates cell cycle checkpoint controls. Proc. Natl. Acad. Sci. U.S.A. 92, 11215–11219. 10.1073/pnas.92.24.11215 7479968 PMC40602

[B17] Berger G. Lawrence M. Hue S. Neil S. J. (2015). G2/M cell cycle arrest correlates with primate lentiviral Vpr interaction with the SLX4 complex. J. Virol. 89, 230–240. 10.1128/JVI.02307-14 25320300 PMC4301105

[B18] Berthet C. Aleem E. Coppola V. Tessarollo L. Kaldis P. (2003). Cdk2 knockout mice are viable. Curr. Biol. 13, 1775–1785. 10.1016/j.cub.2003.09.024 14561402

[B19] Bishop J. M. (1991). Molecular themes in oncogenesis. Cell 64, 235–248. 10.1016/0092-8674(91)90636-D 1988146

[B20] Blagosklonny M. V. Pardee A. B. (2002). The restriction point of the cell cycle. Cell Cycle 1, 103–110. 10.4161/cc.1.2.108 12429916

[B21] Boehm M. Yoshimoto T. Crook M. F. Nallamshetty S. True A. Nabel G. J.. (2002). A growth factor-dependent nuclear kinase phosphorylates p27(Kip1) and regulates cell cycle progression. EMBO J. 21, 3390–3401. 10.1093/emboj/cdf343 12093740 PMC126092

[B22] Bolton D. L. Barnitz R. A. Sakai K. Lenardo M. J. (2008). 14-3-3 theta binding to cell cycle regulatory factors is enhanced by HIV-1 Vpr. Biol. Direct 3:17. 10.1186/1745-6150-3-17 18445273 PMC2390528

[B23] Bondar T. Kalinina A. Khair L. Kopanja D. Nag A. Bagchi S.. (2006). Cul4A and DDB1 associate with Skp2 to target p27Kip1 for proteolysis involving the COP9 signalosome. Mol. Cell. Biol. 26, 2531–2539. 10.1128/MCB.26.7.2531-2539.2006 16537899 PMC1430311

[B24] Boutell C. Everett R. D. (2003). The herpes simplex virus type 1 (HSV-1) regulatory protein ICP0 interacts with and Ubiquitinates p53. J. Biol. Chem. 278, 36596–36602. 10.1074/jbc.M300776200 12855695

[B25] Boutell C. Sadis S. Everett R. D. (2002). Herpes simplex virus type 1 immediate-early protein ICP0 and is isolated RING finger domain act as ubiquitin E3 ligases *in vitro*. J. Virol. 76, 841–850. 10.1128/JVI.76.2.841-850.2002 11752173 PMC136846

[B26] Braunagel S. C. Parr R. Belyavskyi M. Summers M. D. (1998). Autographa californica nucleopolyhedrovirus infection results in Sf9 cell cycle arrest at G2/M phase. Virology 244, 195–211. 10.1006/viro.1998.9097 9581791

[B27] Bresnahan W. A. Thompson E. A. Albrecht T. (1997). Human cytomegalovirus infection results in altered Cdk2 subcellular localization. J. Gen. Virol. 78, 1993–1997. 10.1099/0022-1317-78-8-1993 9266999

[B28] Brewster C. D. Birkenheuer C. H. Vogt M. B. Quackenbush S. L. Rovnak J. (2011). The retroviral cyclin of walleye dermal sarcoma virus binds cyclin-dependent kinases 3 and 8. Virology 409, 299–307. 10.1016/j.virol.2010.10.022 21067790 PMC3008307

[B29] Brown N. R. Noble M. E. Lawrie A. M. Morris M. C. Tunnah P. Divita G.. (1999). Effects of phosphorylation of threonine 160 on cyclin-dependent kinase 2 structure and activity. J. Biol. Chem. 274, 8746–8756. 10.1074/jbc.274.13.8746 10085115

[B30] Bulavin D. V. Higashimoto Y. Demidenko Z. N. Meek S. Graves P. Phillips C.. (2003). Dual phosphorylation controls Cdc25 phosphatases and mitotic entry. Nat. Cell Biol. 5, 545–551. 10.1038/ncb994 12766774

[B31] Card G. L. Knowles P. Laman H. Jones N. McDonald N. Q. (2000). Crystal structure of a gamma-herpesvirus cyclin-cdk complex. EMBO J. 19, 2877–2888. 10.1093/emboj/19.12.2877 10856233 PMC203358

[B32] Carrano A. C. Eytan E. Hershko A. Pagano M. (1999). SKP2 is required for ubiquitin-mediated degradation of the CDK inhibitor p27. Nat. Cell Biol. 1, 193–199. 10.1038/12013 10559916

[B33] Cesarman E. Chang Y. Moore P. S. Said J. W. Knowles D. M. (1995). Kaposi's sarcoma-associated herpesvirus-like DNA-sequences in AIDS-related body-cavity-based lymphomas. N. Engl. J. Med. 332, 1186–1191. 10.1056/NEJM199505043321802 7700311

[B34] Chang Y. Cesarman E. Pessin M. S. Lee F. Culpepper J. Knowles D. M.. (1994). Identification of herpesvirus-like DNA-sequences in AIDS-associated Kaposi's-sarcoma. Science 266, 1865–1869. 10.1126/science.7997879 7997879

[B35] Chang Y. Moore P. S. Talbot S. J. Boshoff C. H. Zarkowska T. Godden K.. (1996). Cyclin encoded by KS herpesvirus. Nature 382:410. 10.1038/382410a0 8684480

[B36] Chang Y. Moore P. S. Weiss R. A. (2017). Human oncogenic viruses: nature and discovery. Philos. Trans. R. Soc. Lond. B. Biol. Sci. 372:20160264. 10.1098/rstb.2016.0264 28893931 PMC5597731

[B37] Chen A. Y. Luo Y. Cheng F. Sun Y. Qiu J. (2010). Bocavirus infection induces mitochondrion-mediated apoptosis and cell cycle arrest at G(2)/M phase. J. Virol. 84, 5615–5626. 10.1128/JVI.02094-09 20335259 PMC2876597

[B38] Chen B. J. Leser G. P. Jackson D. Lamb R. A. (2008). The influenza virus M2 protein cytoplasmic tail interacts with the M1 protein and influences virus assembly at the site of virus budding. J. Virol. 82, 10059–10070. 10.1128/JVI.01184-08 18701586 PMC2566248

[B39] Chen S. Paucha E. (1990). Identification of a region of simian virus 40 large T antigen required for cell transformation. J. Virol. 64, 3350–3357. 2161944 10.1128/jvi.64.7.3350-3357.1990 PMC249578

[B40] Chen Z. Knutson E. Kurosky A. Albrecht T. (2001). Degradation of p21cip1 in cells productively infected with human cytomegalovirus. J. Virol. 75, 3613–3625. 10.1128/JVI.75.8.3613-3625.2001 11264351 PMC114853

[B41] Chiu H. C. Huang W. R. Liao T. L. Chi P. I. Nielsen B. L. Liu J. H. (2018). Mechanistic insights into avian reovirus p17-modulated suppression of cell-cycle CDK/cyclin complexes and enhancement of p53 and cyclin H interaction. J. Biol. Chem. 293, 12542–12562. 10.1074/jbc.RA118.002341 29907572 PMC6093226

[B42] Chiu H. C. Huang W. R. Liao T. L. Wu H. Y. Munir M. Shih W. L.. (2016). Suppression of vimentin phosphorylation by the avian reovirus p17 through inhibition of CDK1 and Plk1 impacting the G2/M phase of the cell cycle. PLoS ONE 11:e0162356. 10.1371/journal.pone.0162356 27603133 PMC5014334

[B43] Choudhuri T. Verma S. C. Lan K. Murakami M. Robertson E. S. (2007). The ATM/ATR signaling effector Chk2 is targeted by Epstein-Barr virus nuclear antigen 3C to release the G(2)/M cell cycle block. J. Virol. 81, 6718–6730. 10.1128/JVI.00053-07 17409144 PMC1900119

[B44] Clark E. Santiago F. Deng L. W. Chong S. Y. de la Fuente C. Wang L.. (2000). Loss of G(1)/S checkpoint in human immunodeficiency virus type 1-infected cells is associated with a lack of cyclin-dependent kinase inhibitor p21/Waf1. J. Virol. 74, 5040–5052. 10.1128/JVI.74.11.5040-5052.2000 10799578 PMC110856

[B45] Conaway R. C. Conaway J. W. (2011). Function and regulation of the Mediator complex. Curr. Opin. Genet. Dev. 21, 225–230. 10.1016/j.gde.2011.01.013 21330129 PMC3086004

[B46] Cortez D. Guntuku S. Qin J. Elledge S. J. (2001). ATR and ATRIP: partners in checkpoint signaling. Science 294, 1713–1716. 10.1126/science.1065521 11721054

[B47] Dang J. Tiwari S. K. Lichinchi G. Qin Y. Patil V. S. Eroshkin A. M.. (2016). Zika virus depletes neural progenitors in human cerebral organoids through activation of the innate immune receptor TLR3. Cell Stem Cell 19, 258–265. 10.1016/j.stem.2016.04.014 27162029 PMC5116380

[B48] Das S. Basu A. (2008). Japanese encephalitis virus infects neural progenitor cells and decreases their proliferation. J. Neurochem. 106, 1624–1636. 10.1111/j.1471-4159.2008.05511.x 18540995

[B49] de Araújo T. V. B. Ximenes R. A. A. Miranda-Filho D. B. Souza W. V. Montarroyos U. R. de Melo A. P. L.. (2018). Association between microcephaly, Zika virus infection, and other risk factors in Brazil: final report of a case-control study. Lancet Infect. Dis. 18, 328–336. 10.1016/S1473-3099(17)30727-2 29242091 PMC7617036

[B50] de Noronha C. M. Sherman M. P. Lin H. W. Cavrois M. V. Moir R. D. Goldman R. D.. (2001). Dynamic disruptions in nuclear envelope architecture and integrity induced by HIV-1 Vpr. Science 294, 1105–1108. 10.1126/science.1063957 11691994

[B51] DeCaprio J. A. Ludlow J. W. Figge J. Shew J. Y. Huang C. M. Lee W. H.. (1988). SV40 large tumor antigen forms a specific complex with the product of the retinoblastoma susceptibility gene. Cell 54, 275–283. 10.1016/0092-8674(88)90559-4 2839300

[B52] Deng L. Nagano-Fujii M. Tanaka M. Nomura-Takigawa Y. Ikeda M. Kato N.. (2006). NS3 protein of Hepatitis C virus associates with the tumour suppressor p53 and inhibits its function in an NS3 sequence-dependent manner. J. Gen. Virol. 87, 1703–1713. 10.1099/vir.0.81735-0 16690937

[B53] Desai D. Gu Y. Morgan D. O. (1992). Activation of human cyclin-dependent kinases *in vitro*. Mol. Biol. Cell 3, 571–582. 10.1091/mbc.3.5.571 1535244 PMC275609

[B54] Devault A. Martinez A. M. Fesquet D. Labbe J. C. Morin N. Tassan J. P.. (1995). MAT1 ('menage a trois') a new RING finger protein subunit stabilizing cyclin H-cdk7 complexes in starfish and Xenopus, CAK. EMBO J. 14, 5027–5036. 10.1002/j.1460-2075.1995.tb00185.x 7588631 PMC394606

[B55] Diehl J. A. Cheng M. Roussel M. F. Sherr C. J. (1998). Glycogen synthase kinase-3beta regulates cyclin D1 proteolysis and subcellular localization. Genes Dev. 12, 3499–3511. 10.1101/gad.12.22.3499 9832503 PMC317244

[B56] Dove B. Brooks G. Bicknell K. Wurm T. Hiscox J. A. (2006). Cell cycle perturbations induced by infection with the coronavirus infectious bronchitis virus and their effect on virus replication. J. Virol. 80, 4147–4156. 10.1128/JVI.80.8.4147-4156.2006 16571830 PMC1440480

[B57] Dubois B. Lamy P. J. Chemin K. Lachaux A. Kaiserlian D. (2001). Measles virus exploits dendritic cells to suppress CD4(+) T-cell proliferation via expression of surface viral glycoproteins independently of T-cell trans-infection. Cell. Immunol. 214, 173–183. 10.1006/cimm.2001.1898 12088416

[B58] Durkin S. S. Guo X. Fryrear K. A. Mihaylova V. T. Gupta S. K. Belgnaoui S. M.. (2008). HTLV-1 Tax oncoprotein subverts the cellular DNA damage response via binding to DNA-dependent protein kinase. J. Biol. Chem. 283, 36311–36320. 10.1074/jbc.M804931200 18957425 PMC2605996

[B59] Dyson N. Howley P. M. Munger K. Harlow E. (1989). The human papilloma virus-16 E7 oncoprotein is able to bind to the retinoblastoma gene product. Science 243, 934–937. 10.1126/science.2537532 2537532

[B60] el-Deiry W. S. Tokino T. Velculescu V. E. Levy D. B. Parsons R. Trent J. M.. (1993). WAF1, a potential mediator of p53 tumor suppression. Cell 75, 817–825. 10.1016/0092-8674(93)90500-P 8242752

[B61] Everett R. D. Earnshaw W. C. Findlay J. Lomonte P. (1999). Specific destruction of kinetochore protein CENP-C and disruption of cell division by herpes simplex virus immediate-early protein Vmw110. EMBO J. 18, 1526–1538. 10.1093/emboj/18.6.1526 10075924 PMC1171241

[B62] Ewen M. E. Sluss H. K. Sherr C. J. Matsushime H. Kato J. Livingston D. M. (1993). Functional interactions of the retinoblastoma protein with mammalian D-type cyclins. Cell 73, 487–497. 10.1016/0092-8674(93)90136-E 8343202

[B63] Faizan M. I. Abdullah M. Ali S. Naqvi I. H. Ahmed A. Parveen S. (2016). Zika virus-induced microcephaly and its possible molecular mechanism. Intervirology 59, 152–158. 10.1159/000452950 28081529

[B64] Fan Y. Mok C. K. Chan M. C. Zhang Y. Nal B. Kien F.. (2017). Cell cycle-independent role of cyclin D3 in host restriction of influenza virus infection. J. Biol. Chem. 292, 5070–5088. 10.1074/jbc.M117.776112 28130444 PMC5377818

[B65] Fekairi S. Scaglione S. Chahwan C. Taylor E. R. Tissier A. Coulon S.. (2009). Human SLX4 is a Holliday junction resolvase subunit that binds multiple DNA repair/recombination endonucleases. Cell 138, 78–89. 10.1016/j.cell.2009.06.029 19596236 PMC2861413

[B66] Fickenscher H. Fleckenstein B. (2001). Herpesvirus saimiri. Philos. Trans. R. Soc. Lond. B. Biol. Sci. 356, 545–567. 10.1098/rstb.2000.0780 11313011 PMC1088444

[B67] Fisher R. P. Morgan D. O. (1994). A novel cyclin associates with MO15/CDK7 to form the CDK-activating kinase. Cell 78, 713–724. 10.1016/0092-8674(94)90535-5 8069918

[B68] Fortunato E. A. Spector D. H. (1998). p53 and RPA are sequestered in viral replication centers in the nuclei of cells infected with human cytomegalovirus. J. Virol. 72, 2033–2039. 9499057 10.1128/jvi.72.3.2033-2039.1998 PMC109496

[B69] Fu H. Subramanian R. R. Masters S. C. (2000). 14-3-3 proteins: structure, function, and regulation. Annu. Rev. Pharmacol. Toxicol. 40, 617–647. 10.1146/annurev.pharmtox.40.1.617 10836149

[B70] Funk J. O. Waga S. Harry J. B. Espling E. Stillman B. Galloway D. A. (1997). Inhibition of CDK activity and PCNA-dependent DNA replication by p21 is blocked by interaction with the HPV-16 E7 oncoprotein. Genes Dev. 11, 2090–2100. 10.1101/gad.11.16.2090 9284048 PMC316456

[B71] Furumoto T. Tanaka A. Ito M. Malik S. Hirose Y. Hanaoka F.. (2007). A kinase subunit of the human mediator complex, CDK8, positively regulates transcriptional activation. Genes Cells 12, 119–132. 10.1111/j.1365-2443.2007.01036.x 17212659

[B72] Gabriel E. Ramani A. Karow U. Gottardo M. Natarajan K. Gooi L. M.. (2017). Recent Zika virus isolates induce premature differentiation of neural progenitors in human brain organoids. Cell Stem Cell 20, 397–406. 10.1016/j.stem.2016.12.005 28132835

[B73] Geng Y. Yu Q. Y. Sicinska E. Das M. Schneider J. E. Bhattacharya S.. (2003). Cyclin E ablation in the mouse. Cell 114, 431–443. 10.1016/S0092-8674(03)00645-7 12941272

[B74] Ghouzzi V. E. Bianchi F. T. Molineris I. Mounce B. C. Berto G. E. Rak M.. (2016). ZIKA virus elicits P53 activation and genotoxic stress in human neural progenitors similar to mutations involved in severe forms of genetic microcephaly. Cell Death Dis. 7:e2440. 10.1038/cddis.2016.266 27787521 PMC5133962

[B75] Glotzer M. (2001). Animal cell cytokinesis. Annu. Rev. Cell Dev. Biol. 17, 351–386. 10.1146/annurev.cellbio.17.1.351 11687493

[B76] Godden-Kent D. Talbot S. J. Boshoff C. Chang Y. Moore P. Weiss R. A.. (1997). The cyclin encoded by Kaposi's sarcoma-associated herpesvirus stimulates cdk6 to phosphorylate the retinoblastoma protein and histone H1. J. Virol. 71, 4193–4198. 9151805 10.1128/jvi.71.6.4193-4198.1997 PMC191633

[B77] Goh W. C. Manel N. Emerman M. (2004). The human immunodeficiency virus Vpr protein binds Cdc25C: implications for G2 arrest. Virology 318, 337–349. 10.1016/j.virol.2003.10.007 14972559

[B78] Gorbsky G. J. (1995). Kinetochores, microtubules and the metaphase checkpoint. Trends Cell Biol. 5, 143–148. 10.1016/S0962-8924(00)88968-0 14732139

[B79] Gottwein E. Cullen B. R. (2010). A human herpesvirus microRNA inhibits p21 expression and attenuates p21-mediated cell cycle arrest. J. Virol. 84, 5229–5237. 10.1128/JVI.00202-10 20219912 PMC2863803

[B80] Gould K. L. Moreno S. Owen D. J. Sazer S. Nurse P. (1991). Phosphorylation at Thr167 is required for Schizosaccharomyces pombe p34cdc2 function. EMBO J. 10, 3297–3309. 10.1002/j.1460-2075.1991.tb04894.x 1655416 PMC453056

[B81] Graves P. R. Lovly C. M. Uy G. L. Piwnica-Worms H. (2001). Localization of human Cdc25C is regulated both by nuclear export and 14-3-3 protein binding. Oncogene 20, 1839–1851. 10.1038/sj.onc.1204259 11313932

[B82] Griffin D. E. (2010). Measles virus-induced suppression of immune responses. Immunol. Rev. 236, 176–189. 10.1111/j.1600-065X.2010.00925.x 20636817 PMC2908915

[B83] Gu Y. Rosenblatt J. Morgan D. O. (1992). Cell cycle regulation of CDK2 activity by phosphorylation of Thr160 and Tyr15. EMBO J. 11, 3995–4005. 10.1002/j.1460-2075.1992.tb05493.x 1396589 PMC556910

[B84] Gulley M. L. Burton M. P. Allred D. C. Nicholls J. M. Amin M. B. Ro J. Y.. (1998). Epstein-Barr virus infection is associated with p53 accumulation in nasopharyngeal carcinoma. Hum. Pathol. 29, 252–259. 10.1016/S0046-8177(98)90044-2 9496828

[B85] Guo L. Tang M. Yang L. Xiao L. Bode A. M. Li L.. (2012). Epstein-Barr virus oncoprotein LMP1 mediates survivin upregulation by p53 contributing to G1/S cell cycle progression in nasopharyngeal carcinoma. Int. J. Mol. Med. 29, 574–580. 10.3892/ijmm.2012.889 22266808 PMC3573768

[B86] Gupta S. K. Guo X. Durkin S. S. Fryrear K. F. Ward M. D. Semmes O. J. (2007). Human T-cell leukemia virus type 1 Tax oncoprotein prevents DNA damage-induced chromatin egress of hyperphosphorylated Chk2. J. Biol. Chem. 282, 29431–29440. 10.1074/jbc.M704110200 17698850

[B87] Hagglund R. Van Sant C. Lopez P. Roizman B. (2002). Herpes simplex virus 1-infected cell protein 0 contains two E3 ubiquitin ligase sites specific for different E2 ubiquitin-conjugating enzymes. Proc. Natl. Acad. Sci. U.S.A. 99, 631–636. 10.1073/pnas.022531599 11805320 PMC117357

[B88] Haller K. Wu Y. L. Derow E. Schmitt I. Jeang K. T. Grassmann R. (2002). Physical interaction of human T-cell leukemia virus type 1 Tax with cyclin-dependent kinase 4 stimulates the phosphorylation of retinoblastoma protein. Mol. Cell. Biol. 22, 3327–3338. 10.1128/MCB.22.10.3327-3338.2002 11971966 PMC133776

[B89] Haoudi A. Daniels R. C. Wong E. Kupfer G. Semmes O. J. (2003). Human T-cell leukemia virus-I tax oncoprotein functionally targets a subnuclear complex involved in cellular DNA damage-response. J. Biol. Chem. 278, 37736–37744. 10.1074/jbc.M301649200 12842897

[B90] Hartwell L. H. Weinert T. A. (1989). Checkpoints: controls that ensure the order of cell cycle events. Science 246, 629–634. 10.1126/science.2683079 2683079

[B91] He Y. A. Xu K. Keiner B. Zhou J. F. Czudai V. Li T. X. (2010). Influenza A virus replication induces cell cycle arrest in G(0)/G(1) phase. J. Virol. 84, 12832–12840. 10.1128/JVI.01216-10 20861262 PMC3004346

[B92] Hirao A. Kong Y. Y. Matsuoka S. Wakeham A. Ruland J. Yoshida H.. (2000). DNA damage-induced activation of p53 by the checkpoint kinase Chk2. Science 287, 1824–1827. 10.1126/science.287.5459.1824 10710310

[B93] Hobbs W. E. II. DeLuca N. A. (1999). Perturbation of cell cycle progression and cellular gene expression as a function of herpes simplex virus ICP0. J. Virol. 73, 8245–8255. 10482575 10.1128/jvi.73.10.8245-8255.1999 PMC112842

[B94] Hoen B. Schaub B. Funk A. L. Ardillon V. Boullard M. Cabie A.. (2018). Pregnancy outcomes after ZIKV infection in French Territories in the Americas. N. Engl. J. Med. 378, 985–994. 10.1056/NEJMoa1709481 29539287

[B95] Hoffman W. H. Biade S. Zilfou J. T. Chen J. Murphy M. (2002). Transcriptional repression of the anti-apoptotic survivin gene by wild type p53. J. Biol. Chem. 277, 3247–3257. 10.1074/jbc.M106643200 11714700

[B96] Hsu J. Y. Reimann J. D. R. Sorensen C. S. Lukas J. Jackson P. K. (2002). E2F-dependent accumulation of hEmi1 regulates S phase entry by inhibiting APC(Cdh1). Nat. Cell Biol. 4, 358–366. 10.1038/ncb785 11988738

[B97] Huang P. S. Patrick D. R. Edwards G. Goodhart P. J. Huber H. E. Miles L.. (1993). Protein domains governing interactions between E2F, the retinoblastoma gene product, and human papillomavirus type 16 E7 protein. Mol. Cell. Biol. 13, 953–960. 10.1128/MCB.13.2.953 7678696 PMC358979

[B98] Huang W. R. Chi P. I. Chiu H. C. Hsu J. L. Nielsen B. L. Liao T. L.. (2017). Avian reovirus p17 and sigmaA act cooperatively to downregulate Akt by suppressing mTORC2 and CDK2/cyclin A2 and upregulating proteasome PSMB6. Sci. Rep. 7:5226. 10.1038/s41598-017-05510-x 28701787 PMC5507987

[B99] Huang W. R. Chiu H. C. Liao T. L. Chuang K. P. Shih W. L. Liu H. J. (2015). Avian reovirus protein p17 functions as a nucleoporin Tpr suppressor leading to activation of p53, p21 and PTEN and inactivation of PI3K/AKT/mTOR and ERK signaling pathways. PLoS One. 10:e0138627 10.1371/journal.pone.0138627 26368931 PMC4569338

[B100] Huang X. D. Summers M. K. Pham V. Lill J. R. Liu J. F. Lee G.. (2011). Deubiquitinase USP37 is activated by CDK2 to antagonize APC(CDH1) and promote S phase entry. Mol. Cell 42, 511–523. 10.1016/j.molcel.2011.03.027 21596315

[B101] Huard S. Elder R. T. Liang D. Li G. Zhao R. Y. (2008). Human immunodeficiency virus type 1 vpr induces cell cycle G(2) arrest through Srk1/MK2-mediated phosphorylation of Cdc25. J. Virol. 82, 2904–2917. 10.1128/JVI.01098-07 18160429 PMC2259012

[B102] Hume A. J. Finkel J. S. Kamil J. P. Coen D. M. Culbertson M. R. Kalejta R. F. (2008). Phosphorylation of retinoblastoma protein by viral protein with cyclin-dependent kinase function. Science 320, 797–799. 10.1126/science.1152095 18467589

[B103] Hunt T. (1989). Maturation promoting factor, cyclin and the control of M-phase. Curr. Opin. Cell Biol. 1, 268–274. 10.1016/0955-0674(89)90099-9 2576632

[B104] Ikeda M. A. Nevins J. R. (1993). Identification of distinct roles for separate E1A domains in disruption of E2F complexes. Mol. Cell. Biol. 13, 7029–7035. 10.1128/MCB.13.11.7029 8413292 PMC364764

[B105] Inman G. J. Farrell P. J. (1995). Epstein-Barr virus EBNA-LP and transcription regulation properties of pRB, p107 and p53 in transfection assays. J. Gen. Virol. 76, 2141–2149. 10.1099/0022-1317-76-9-2141 7561751

[B106] Iovine B. Iannella M. L. Bevilacqua M. A. (2011). Damage-specific DNA binding protein 1 (DDB1): a protein with a wide range of functions. Int. J. Biochem. Cell Biol. 43, 1664–1667. 10.1016/j.biocel.2011.09.001 21959250

[B107] Irniger S. Piatti S. Michaelis C. Nasmyth K. (1995). Genes involved in sister chromatid separation are needed for B-type cyclin proteolysis in budding yeast. Cell 81, 269–278. 10.1016/0092-8674(95)90337-2 7736579

[B108] Ishida N. Hara T. Kamura T. Yoshida M. Nakayama K. Nakayama K. I. (2002). Phosphorylation of p27Kip1 on serine 10 is required for its binding to CRM1 and nuclear export. J. Biol. Chem. 277, 14355–14358. 10.1074/jbc.C100762200 11889117

[B109] Ishido S. Hotta H. (1998). Complex formation of the nonstructural protein 3 of hepatitis C virus with the p53 tumor suppressor. FEBS Lett. 438, 258–262. 10.1016/S0014-5793(98)01312-X 9827557

[B110] Iwanaga R. Ozono E. Fujisawa J. Ikeda M. A. Okamura N. Huang Y.. (2008). Activation of the cyclin D2 and cdk6 genes through NF-kappaB is critical for cell-cycle progression induced by HTLV-I Tax. Oncogene 27, 5635–5642. 10.1038/onc.2008.174 18504428

[B111] Izumi T. Io K. Matsui M. Shirakawa K. Shinohara M. Nagai Y.. (2010). HIV-1 viral infectivity factor interacts with TP53 to induce G2 cell cycle arrest and positively regulate viral replication. Proc. Natl. Acad. Sci. U.S.A. 107, 20798–20803. 10.1073/pnas.1008076107 21071676 PMC2996458

[B112] Izumi T. Takaori-Kondo A. Shirakawa K. Higashitsuji H. Itoh K. Io K.. (2009). MDM2 is a novel E3 ligase for HIV-1 Vif. Retrovirology 6:1. 10.1186/1742-4690-6-1 19128510 PMC2629459

[B113] Izumiya Y. Lin S. F. Ellison T. J. Levy A. M. Mayeur G. L. Izumiya C. (2003). Cell cycle regulation by Kaposi's sarcoma-associated herpesvirus K-bZIP: Direct interaction with cyclin-CDK2 and induction of G(1) growth arrest. J. Virol. 77, 9652–9661. 10.1128/JVI.77.17.9652-9661.2003 12915577 PMC187423

[B114] Jiang W. Wang Q. Chen S. Gao S. Song L. Liu P.. (2013). Influenza A virus NS1 induces G0/G1 cell cycle arrest by inhibiting the expression and activity of RhoA protein. J. Virol. 87, 3039–3052. 10.1128/JVI.03176-12 23283961 PMC3592114

[B115] Jiang W. Q. Szekely L. Wendel-Hansen V. Ringertz N. Klein G. Rosen A. (1991). Co-localization of the retinoblastoma protein and the Epstein-Barr virus-encoded nuclear antigen EBNA-5. Exp. Cell Res. 197, 314–318. 10.1016/0014-4827(91)90438-Z 1659990

[B116] Jin P. Gu Y. Morgan D. O. (1996). Role of inhibitory CDC2 phosphorylation in radiation-induced G2 arrest in human cells. J. Cell Biol. 134, 963–970. 10.1083/jcb.134.4.963 8769420 PMC2120957

[B117] Johnson R. A. Yurochko A. D. Poma E. E. Zhu L. Huang E. S. (1999). Domain mapping of the human cytomegalovirus IE1-72 and cellular p107 protein-protein interaction and the possible functional consequences. J. Gen. Virol. 80, 1293–1303. 10.1099/0022-1317-80-5-1293 10355776

[B118] Jones D. L. Alani R. M. Munger K. (1997). The human papillomavirus E7 oncoprotein can uncouple cellular differentiation and proliferation in human keratinocytes by abrogating p21Cip1-mediated inhibition of cdk2. Genes Dev. 11, 2101–2111. 10.1101/gad.11.16.2101 9284049 PMC316455

[B119] Jung J. U. Stager M. Desrosiers R. C. (1994). Virus-encoded cyclin. Mol. Cell. Biol. 14, 7235–7244. 10.1128/MCB.14.11.7235 7935438 PMC359258

[B120] Kaldis P. (1999). The cdk-activating kinase (CAK): from yeast to mammals. Cell. Mol. Life Sci. 55, 284–296. 10.1007/s000180050290 10188587 PMC11146862

[B121] Kaldis P. Russo A. A. Chou H. S. Pavletich N. P. Solomon M. J. (1998). Human and yeast cdk-activating kinases (CAKs) display distinct substrate specificities. Mol. Biol. Cell 9, 2545–2560. 10.1091/mbc.9.9.2545 9725911 PMC25525

[B122] Kamata M. Watanabe N. Nagaoka Y. Chen I. S. (2008). Human immunodeficiency virus type 1 Vpr binds to the N lobe of the Wee1 kinase domain and enhances kinase activity for CDC2. J. Virol. 82, 5672–5682. 10.1128/JVI.01330-07 18385244 PMC2395134

[B123] Kato J. Y. Matsuoka M. Strom D. K. Sherr C. J. (1994). Regulation of cyclin D-dependent kinase 4 (cdk4) by cdk4-activating kinase. Mol. Cell. Biol. 14, 2713–2721. 10.1128/MCB.14.4.2713 8139570 PMC358637

[B124] Kim J. Y. Park S. Y. Lyoo H. R. Koo E. S. Kim M. S. Jeong Y. S. (2015). Extended stability of cyclin D1 contributes to limited cell cycle arrest at G1-phase in BHK-21 cells with Japanese encephalitis virus persistent infection. J. Microbiol. 53, 77–83. 10.1007/s12275-015-4661-z 25557483 PMC7090764

[B125] Kim Y. Spitz G. S. Veturi U. Lach F. P. Auerbach A. D. Smogorzewska A. (2013). Regulation of multiple DNA repair pathways by the Fanconi anemia protein SLX4. Blood 121, 54–63. 10.1182/blood-2012-07-441212 23093618 PMC3538331

[B126] King R. W. Peters J. M. Tugendreich S. Rolfe M. Hieter P. Kirschner M. W. (1995). A 20S complex containing CDC27 and CDC16 catalyzes the mitosis-specific conjugation of ubiquitin to cyclin B. Cell 81, 279–288. 10.1016/0092-8674(95)90338-0 7736580

[B127] Kino T. Gragerov A. Valentin A. Tsopanomihalou M. Ilyina-Gragerova G. Erwin-Cohen R.. (2005). Vpr protein of human immunodeficiency virus type 1 binds to 14-3-3 proteins and facilitates complex formation with Cdc25C: implications for cell cycle arrest. J. Virol. 79, 2780–2787. 10.1128/JVI.79.5.2780-2787.2005 15708996 PMC548435

[B128] Knight J. S. Robertson E. S. (2004). Epstein-Barr virus nuclear antigen 3C regulates cyclin A/p27 complexes and enhances cyclin A-dependent kinase activity. J. Virol. 78, 1981–1991. 10.1128/JVI.78.4.1981-1991.2004 14747563 PMC369513

[B129] Kopan R. Ilagan M. X. (2009). The canonical Notch signaling pathway: unfolding the activation mechanism. Cell 137, 216–233. 10.1016/j.cell.2009.03.045 19379690 PMC2827930

[B130] Kovacs A. Weber M. L. Burns L. J. Jacob H. S. Vercellotti G. M. (1996). Cytoplasmic sequestration of p53 in cytomegalovirus-infected human endothelial cells. Am. J. Pathol. 149, 1531–1539. 8909243 PMC1865290

[B131] Kozar K. Ciemerych M. A. Rebel V. I. Shigematsu H. Zagozdzon A. Sicinska E.. (2004). Mouse development and cell proliferation in the absence of D-cyclins. Cell 118, 477–491. 10.1016/j.cell.2004.07.025 15315760

[B132] Krek W. Nigg E. A. (1992). Cell cycle regulation of vertebrate p34cdc2 activity: identification of Thr161 as an essential *in vivo* phosphorylation site. New Biol. 4, 323–329. 1622929

[B133] Krude T. Jackman M. Pines J. Laskey R. A. (1997). Cyclin/Cdk-dependent initiation of DNA replication in a human cell-free system. Cell 88, 109–119. 10.1016/S0092-8674(00)81863-2 9019396

[B134] Kuo Y. L. Giam C. Z. (2006). Activation of the anaphase promoting complex by HTLV-1 tax leads to senescence. EMBO J. 25, 1741–1752. 10.1038/sj.emboj.7601054 16601696 PMC1440834

[B135] Lacey K. R. Jackson P. K. Stearns T. (1999). Cyclin-dependent kinase control of centrosome duplication. Proc. Natl. Acad. Sci. U.S.A. 96, 2817–2822. 10.1073/pnas.96.6.2817 10077594 PMC15852

[B136] Laguette N. Bregnard C. Hue P. Basbous J. Yatim A. Larroque M.. (2014). Premature activation of the SLX4 complex by Vpr promotes G2/M arrest and escape from innate immune sensing. Cell 156, 134–145. 10.1016/j.cell.2013.12.011 24412650

[B137] Lai M. Zimmerman E. S. Planelles V. Chen J. (2005). Activation of the ATR pathway by human immunodeficiency virus type 1 Vpr involves its direct binding to chromatin *in vivo*. J. Virol. 79, 15443–15451. 10.1128/JVI.79.24.15443-15451.2005 16306615 PMC1315993

[B138] Lan K. H. Sheu M. L. Hwang S. J. Yen S. H. Chen S. Y. Wu J. C.. (2002). HCV NS5A interacts with p53 and inhibits p53-mediated apoptosis. Oncogene 21, 4801–4811. 10.1038/sj.onc.1205589 12101418

[B139] Lane D. P. Crawford L. V. (1979). T antigen is bound to a host protein in SV40-transformed cells. Nature 278, 261–263. 10.1038/278261a0 218111

[B140] LaPierre L. A. Casey J. W. Holzschu D. L. (1998). Walleye retroviruses associated with skin tumors and hyperplasias encode cyclin D homologs. J. Virol. 72, 8765–8771. 9765420 10.1128/jvi.72.11.8765-8771.1998 PMC110292

[B141] Laronga C. Yang H. Y. Neal C. Lee M. H. (2000). Association of the cyclin-dependent kinases and 14-3-3 sigma negatively regulates cell cycle progression. J. Biol. Chem. 275, 23106–23112. 10.1074/jbc.M905616199 10767298

[B142] Le Rouzic E. Belaidouni N. Estrabaud E. Morel M. Rain J.-C. Transy C.. (2007). HIV1 Vpr arrests the cell cycle by recruiting DCAF1/VprBP, a receptor of the Cul4-DDB1 ubiquitin ligase. Cell Cycle 6, 182–188. 10.4161/cc.6.2.3732 17314515

[B143] Le L. Leluk J. (2011). Study on phylogenetic relationships, variability, and correlated mutations in M2 proteins of influenza virus A. PLoS ONE 6:e22970. 10.1371/journal.pone.0022970 21829678 PMC3149066

[B144] Lehman J. M. Laffin J. Friedrich T. D. (2000). Simian virus 40 induces multiple S phases with the majority of viral DNA replication in the G2 and second S phase in CV-1 cells. Exp. Cell Res. 258, 215–222. 10.1006/excr.2000.4927 10912803

[B145] Leupin O. Bontron S. Strubin M. (2003). Hepatitis B virus X protein and simian virus 5 V protein exhibit similar UV-DDB1 binding properties to mediate distinct activities. J. Virol. 77, 6274–6283. 10.1128/JVI.77.11.6274-6283.2003 12743284 PMC154990

[B146] Levine A. J. (1997). p53, the cellular gatekeeper for growth and division. Cell 88, 323–331. 10.1016/S0092-8674(00)81871-1 9039259

[B147] Lew D. J. Dulic V. Reed S. I. (1991). Isolation of three novel human cyclins by rescue of G1 cyclin (Cln) function in yeast. Cell 66, 1197–1206. 10.1016/0092-8674(91)90042-W 1833066

[B148] Li C. Xu D. Ye Q. Hong S. Jiang Y. Liu X. (2016). Zika virus disrupts neural progenitor development and leads to microcephaly in mice. Cell Stem Cell 19, 120–126. 10.1016/j.stem.2016.04.017 27179424

[B149] Li G. Park H. U. Liang D. Zhao R. Y. (2010). Cell cycle G2/M arrest through an S phase-dependent mechanism by HIV-1 viral protein R. Retrovirology 7:59. 10.1186/1742-4690-7-59 20609246 PMC2909154

[B150] Li G. Poulsen M. Fenyvuesvolgyi C. Yashiroda Y. Yoshida M. Simard J. M.. (2017). Characterization of cytopathic factors through genome-wide analysis of the Zika viral proteins in fission yeast. Proc. Natl. Acad. Sci. U.S.A. 114, E376–E385. 10.1073/pnas.1619735114 28049830 PMC5255628

[B151] Li H. Baskaran R. Krisky D. M. Bein K. Grandi P. Cohen J. B.. (2008). Chk2 is required for HSV-1 ICP0-mediated G2/M arrest and enhancement of virus growth. Virology 375, 13–23. 10.1016/j.virol.2008.01.038 18321553 PMC2706573

[B152] Li J. Stern D. F. (2005a). DNA damage regulates Chk2 association with chromatin. J. Biol. Chem. 280, 37948–37956. 10.1074/jbc.M509299200 16150728

[B153] Li J. Stern D. F. (2005b). Regulation of CHK2 by DNA-dependent protein kinase. J. Biol. Chem. 280, 12041–12050. 10.1074/jbc.M412445200 15668230

[B154] Li L. Gu B. Zhou F. Chi J. Wang F. Peng G.. (2011). Human herpesvirus 6 suppresses T cell proliferation through induction of cell cycle arrest in infected cells in the G2/M phase. J. Virol. 85, 6774–6783. 10.1128/JVI.02577-10 21525341 PMC3126536

[B155] Li M. Lee H. Yoon D. W. Albrecht J. C. Fleckenstein B. Neipel F.. (1997). Kaposi's sarcoma-associated herpesvirus encodes a functional cyclin. J. Virol. 71, 1984–1991. 9032330 10.1128/jvi.71.3.1984-1991.1997 PMC191282

[B156] Liao J. B. (2006). Viruses and human cancer. Yale J. Biol. Med. 79, 115–122. 17940621 PMC1994798

[B157] Lin P. Y. Lee J. W. Liao M. H. Hsu H. Y. Chiu S. J. Liu H. J.. (2009). Modulation of p53 by mitogen-activated protein kinase pathways and protein kinase C delta during avian reovirus S1133-induced apoptosis. Virology 385, 323–334. 10.1016/j.virol.2008.12.028 19168198

[B158] Linzer D. I. Levine A. J. (1979). Characterization of a 54K dalton cellular SV40 tumor antigen present in SV40-transformed cells and uninfected embryonal carcinoma cells. Cell 17, 43–52. 10.1016/0092-8674(79)90293-9 222475

[B159] Liu B. Hong S. Tang Z. Yu H. Giam C. Z. (2005). HTLV-I Tax directly binds the Cdc20-associated anaphase-promoting complex and activates it ahead of schedule. Proc. Natl. Acad. Sci. U.S.A. 102, 63–68. 10.1073/pnas.0406424101 15623561 PMC544051

[B160] Liu B. Liang M. H. Kuo Y. L. Liao W. Boros I. Kleinberger T.. (2003). Human T-lymphotropic virus type 1 oncoprotein tax promotes unscheduled degradation of Pds1p/securin and Clb2p/cyclin B1 and causes chromosomal instability. Mol. Cell. Biol. 23, 5269–5281. 10.1128/MCB.23.15.5269-5281.2003 12861013 PMC165734

[B161] Liu J. Li Q. Li X. Qiu Z. Li A. Liang W.. (2018). Zika Virus Envelope Protein induces G2/M cell cycle arrest and apoptosis via an intrinsic cell death signaling pathway in neuroendocrine PC12 cells. Int. J. Biol. Sci. 14, 1099–1108. 10.7150/ijbs.26400 29989100 PMC6036729

[B162] Lohka M. J. Hayes M. K. Maller J. L. (1988). Purification of maturation-promoting factor, an intracellular regulator of early mitotic events. Proc. Natl. Acad. Sci. U.S.A. 85, 3009–3013. 10.1073/pnas.85.9.3009 3283736 PMC280132

[B163] Lomonte P. Sullivan K. F. Everett R. D. (2001). Degradation of nucleosome-associated centromeric histone H3-like protein CENP-A induced by herpes simplex virus type 1 protein ICP0. J. Biol. Chem. 276, 5829–5835. 10.1074/jbc.M008547200 11053442

[B164] López-Avilés S. Grande M. Gonzalez M. Helgesen A. L. Alemany V. Sanchez-Piris M.. (2005). Inactivation of the Cdc25 phosphatase by the stress-activated Srk1 kinase in fission yeast. Mol. Cell 17, 49–59. 10.1016/j.molcel.2004.11.043 15629716

[B165] Lopez-Girona A. Furnari B. Mondesert O. Russell P. (1999). Nuclear localization of Cdc25 is regulated by DNA damage and a 14-3-3 protein. Nature 397, 172–175. 10.1038/16488 9923681

[B166] Lopez-Girona A. Tanaka K. Chen X. B. Baber B. A. McGowan C. H. Russell P. (2001). Serine-345 is required for Rad3-dependent phosphorylation and function of checkpoint kinase Chk1 in fission yeast. Proc. Natl. Acad. Sci. U.S.A. 98, 11289–11294. 10.1073/pnas.191557598 11553781 PMC58722

[B167] Low K. G. Dorner L. F. Fernando D. B. Grossman J. Jeang K. T. Comb M. J. (1997). Human T-cell leukemia virus type 1 Tax releases cell cycle arrest induced by p16INK4a. J. Virol. 71, 1956–1962. 9032327 10.1128/jvi.71.3.1956-1962.1997 PMC191279

[B168] Lowe M. Rabouille C. Nakamura N. Watson R. Jackman M. Jamsa E.. (1998). Cdc2 kinase directly phosphorylates the cis-Golgi matrix protein GM130 and is required for Golgi fragmentation in mitosis. Cell 94, 783–793. 10.1016/S0092-8674(00)81737-7 9753325

[B169] Lu H. Levine A. J. (1995). Human TAFII31 protein is a transcriptional coactivator of the p53 protein. Proc. Natl. Acad. Sci. U.S.A. 92, 5154–5158. 10.1073/pnas.92.11.5154 7761466 PMC41867

[B170] Ma H. Kien F. Maniere M. Zhang Y. Lagarde N. Tse K. S.. (2012). Human annexin A6 interacts with influenza A virus M2 protein and negatively modulates infection. J. Virol. 86, 1789–1801. 10.1128/JVI.06003-11 22114333 PMC3264383

[B171] Mahony D. Parry D. A. Lees E. (1998). Active cdk6 complexes are predominantly nuclear and represent only a minority of the cdk6 in T cells. Oncogene 16, 603–611. 10.1038/sj.onc.1201570 9482106

[B172] Majumder M. Ghosh A. K. Steele R. Ray R. Ray R. B. (2001). Hepatitis C virus NS5A physically associates with p53 and regulates p21/waf1 gene expression in a p53-dependent manner. J. Virol. 75, 1401–1407. 10.1128/JVI.75.3.1401-1407.2001 11152513 PMC114046

[B173] Mal A. Poon R. Y. Howe P. H. Toyoshima H. Hunter T. Harter M. L. (1996). Inactivation of p27Kip1 by the viral E1A oncoprotein in TGFbeta-treated cells. Nature 380, 262–265. 10.1038/380262a0 8637577

[B174] Malumbres M. (2014). Cyclin-dependent kinases. Genome Biol. 15:122. 10.1186/gb4184 25180339 PMC4097832

[B175] Malumbres M. Sotillo R. Santamaria D. Galan J. Cerezo A. Ortega S.. (2004). Mammalian cells cycle without the D-type cyclin-dependent kinases Cdk4 and Cdk6. Cell 118, 493–504. 10.1016/j.cell.2004.08.002 15315761

[B176] Manke I. A. Nguyen A. Lim D. Stewart M. Q. Elia A. E. Yaffe M. B. (2005). MAPKAP kinase-2 is a cell cycle checkpoint kinase that regulates the G2/M transition and S phase progression in response to UV irradiation. Mol. Cell 17, 37–48. 10.1016/j.molcel.2004.11.021 15629715

[B177] Mann D. J. Child E. S. Swanton C. Laman H. Jones N. (1999). Modulation of p27(Kip1) levels by the cyclin encoded by Kaposi's sarcoma-associated herpesvirus. EMBO J. 18, 654–663. 10.1093/emboj/18.3.654 9927425 PMC1171158

[B178] Mannick J. B. Cohen J. I. Birkenbach M. Marchini A. Kieff E. (1991). The Epstein-Barr virus nuclear protein encoded by the leader of the EBNA RNAs is important in B-lymphocyte transformation. J. Virol. 65, 6826–6837. 1658376 10.1128/jvi.65.12.6826-6837.1991 PMC250776

[B179] Martin-Lluesma S. Schaeffer C. Robert E. I. van Brengel P. C. Leupin O. Hantz O.. (2008). Hepatitis B virus X protein affects S phase progression leading to chromosome segregation defects by binding to damaged DNA binding protein 1. Hepatology 48, 1467–1476. 10.1002/hep.22542 18781669

[B180] Matsuoka M. Kato J. Y. Fisher R. P. Morgan D. O. Sherr C. J. (1994). Activation of cyclin-dependent kinase 4 (cdk4) by mouse MO15-associated kinase. Mol. Cell. Biol. 14, 7265–7275. 10.1128/MCB.14.11.7265 7935441 PMC359261

[B181] Matsuoka S. Huang M. Elledge S. J. (1998). Linkage of ATM to cell cycle regulation by the Chk2 protein kinase. Science 282, 1893–1897. 10.1126/science.282.5395.1893 9836640

[B182] Matsushime H. Quelle D. E. Shurtleff S. A. Shibuya M. Sherr C. J. Kato J. Y. (1994). D-type cyclin-dependent kinase activity in mammalian cells. Mol. Cell. Biol. 14, 2066–2076. 10.1128/MCB.14.3.2066 8114738 PMC358567

[B183] Mayo L. D. Donner D. B. (2002). The PTEN, Mdm2, p53 tumor suppressor-oncoprotein network. Trends Biochem. Sci. 27, 462–467. 10.1016/S0968-0004(02)02166-7 12217521

[B184] McGowan C. H. Russell P. (1993). Human Wee1 kinase inhibits cell division by phosphorylating p34cdc2 exclusively on Tyr15. EMBO J. 12, 75–85. 10.1002/j.1460-2075.1993.tb05633.x 8428596 PMC413177

[B185] Mesri E. A. Feitelson M. A. Munger K. (2014). Human viral oncogenesis: a cancer hallmarks analysis. Cell Host Microbe 15, 266–282. 10.1016/j.chom.2014.02.011 24629334 PMC3992243

[B186] Mietz J. A. Unger T. Huibregtse J. M. Howley P. M. (1992). The transcriptional transactivation function of wild-type-p53 is inhibited by SV40 large T-antigen and by Hpv-16 E6-oncoprotein. EMBO J. 11, 5013–5020. 10.1002/j.1460-2075.1992.tb05608.x 1464323 PMC556979

[B187] Millar J. B. Russell P. (1992). The cdc25 M-phase inducer: an unconventional protein phosphatase. Cell 68, 407–410. 10.1016/0092-8674(92)90177-E 1310893

[B188] Mirza A. McGuirk M. Hockenberry T. N. Wu Q. Ashar H. Black S.. (2002). Human survivin is negatively regulated by wild-type p53 and participates in p53-dependent apoptotic pathway. Oncogene 21, 2613–2622. 10.1038/sj.onc.1205353 11965534

[B189] Mitra J. Enders G. H. Azizkhan-Clifford J. Lengel K. L. (2006). Dual regulation of the anaphase promoting complex in human cells by cyclin A-Cdk2 and cyclin A-Cdk1 complexes. Cell Cycle 5, 661–666. 10.4161/cc.5.6.2604 16582612

[B190] Mlechkovich G. Frenkel N. (2007). Human herpesvirus 6A (HHV-6A) and HHV-6B alter E2F1/Rb pathways and E2F1 localization and cause cell cycle arrest in infected T cells. J. Virol. 81, 13499–13508. 10.1128/JVI.01496-07 17913805 PMC2168879

[B191] Mo M. Fleming S. B. Mercer A. A. (2009). Cell cycle deregulation by a poxvirus partial mimic of anaphase-promoting complex subunit 11. Proc. Natl. Acad. Sci. U.S.A. 106, 19527–19532. 10.1073/pnas.0905893106 19887645 PMC2780751

[B192] Moir R. D. Spann T. P. Herrmann H. Goldman R. D. (2000). Disruption of nuclear lamin organization blocks the elongation phase of DNA replication. J. Cell Biol. 149, 1179–1192. 10.1083/jcb.149.6.1179 10851016 PMC2175110

[B193] Momand J. Wu H. H. Dasgupta G. (2000). MDM2–master regulator of the p53 tumor suppressor protein. Gene 242, 15–29. 10.1016/S0378-1119(99)00487-4 10721693

[B194] Moore P. S. Chang Y. (1998). Antiviral activity of tumor-suppressor pathways: clues from molecular piracy by KSHV. Trends Genet. 14, 144–150. 10.1016/S0168-9525(98)01408-5 9594662

[B195] Morris M. C. Gondeau C. Tainer J. A. Divita G. (2002). Kinetic mechanism of activation of the Cdk2/cyclin A complex. Key role of the C-lobe of the Cdk. J. Biol. Chem. 277, 23847–23853. 10.1074/jbc.M107890200 11959850

[B196] Mukherji A. Janbandhu V. C. Kumar V. (2007). HBx-dependent cell cycle deregulation involves interaction with cyclin E/A-cdk2 complex and destabilization of p27Kip1. Biochem. J. 401, 247–256. 10.1042/BJ20061091 16939421 PMC1698683

[B197] Muñoz I. M. Hain K. Declais A. C. Gardiner M. Toh G. W. Sanchez-Pulido L.. (2009). Coordination of structure-specific nucleases by human SLX4/BTBD12 is required for DNA repair. Mol. Cell 35, 116–127. 10.1016/j.molcel.2009.06.020 19595721

[B198] Muralidhar S. Doniger J. Mendelson E. Araujo J. C. Kashanchi F. Azumi N.. (1996). Human cytomegalovirus mtrII oncoprotein binds to p53 and down-regulates p53-activated transcription. J. Virol. 70, 8691–8700. 8970996 10.1128/jvi.70.12.8691-8700.1996 PMC190964

[B199] Muslin A. J. Xing H. (2000). 14-3-3 proteins: regulation of subcellular localization by molecular interference. Cell. Signal. 12, 703–709. 10.1016/S0898-6568(00)00131-5 11152955

[B200] Nakayama K. Nagahama H. Minamishima Y. A. Miyake S. Ishida N. Hatakeyama S.. (2004). Skp2-mediated degradation of p27 regulates progression into mitosis. Dev. Cell 6, 661–672. 10.1016/S1534-5807(04)00131-5 15130491

[B201] Nascimento R. Dias J. D. Parkhouse R. M. (2009). The conserved UL24 family of human alpha, beta and gamma herpesviruses induces cell cycle arrest and inactivation of the cyclinB/cdc2 complex. Arch. Virol. 154, 1143–1149. 10.1007/s00705-009-0420-y 19526192

[B202] Nascimento R. Parkhouse R. M. E. (2007). Murine gammaherpesvirus 68 ORF20 induces cell-cycle arrest in G(2) by inhibiting the Cdc2-cyclin B complex. J. Gen. Virol. 88, 1446–1453. 10.1099/vir.0.82589-0 17412972

[B203] Nasmyth K. Peters J. M. Uhlmann F. (2000). Splitting the chromosome: cutting the ties that bind sister chromatids. Science 288, 1379–1385. 10.1126/science.288.5470.1379 10827941

[B204] Neuveut C. Low K. G. Maldarelli F. Schmitt I. Majone F. Grassmann R.. (1998). Human T-cell leukemia virus type 1 Tax and cell cycle progression: role of cyclin D-cdk and p110Rb. Mol. Cell. Biol. 18, 3620–3632. 10.1128/MCB.18.6.3620 9584203 PMC108944

[B205] Nicholas J. Cameron K. R. Honess R. W. (1992). Herpesvirus saimiri encodes homologues of G protein-coupled receptors and cyclins. Nature 355, 362–365. 10.1038/355362a0 1309943

[B206] Nigg E. A. (1995). Cyclin-dependent protein kinases: key regulators of the eukaryotic cell cycle. Bioessays 17, 471–480. 10.1002/bies.950170603 7575488

[B207] Norbury C. Blow J. Nurse P. (1991). Regulatory phosphorylation of the p34cdc2 protein kinase in vertebrates. EMBO J. 10, 3321–3329. 10.1002/j.1460-2075.1991.tb04896.x 1655417 PMC453058

[B208] Nurse P. (1990). Universal control mechanism regulating onset of M-phase. Nature 344, 503–508. 10.1038/344503a0 2138713

[B209] Ohtani K. DeGregori J. Nevins J. R. (1995). Regulation of the cyclin E gene by transcription factor E2F1. Proc. Natl. Acad. Sci. U.S.A. 92, 12146–12150. 10.1073/pnas.92.26.12146 8618861 PMC40313

[B210] Ooi M. H. Solomon T. Podin Y. Mohan A. Akin W. Yusuf M. A.. (2007). Evaluation of different clinical sample types in diagnosis of human enterovirus 71-associated hand-foot-and-mouth disease. J. Clin. Microbiol. 45, 1858–1866. 10.1128/JCM.01394-06 17446325 PMC1933032

[B211] Op De Beeck A. Anouja F. Mousset S. Rommelaere J. Caillet-Fauquet P. (1995). The nonstructural proteins of the autonomous parvovirus minute virus of mice interfere with the cell cycle, inducing accumulation in G2. Cell Growth Differ. 6, 781–787. 7547499

[B212] Op De Beeck A. Caillet-Fauquet P. (1997). The NS1 protein of the autonomous parvovirus minute virus of mice blocks cellular DNA replication: a consequence of lesions to the chromatin? J. Virol. 71, 5323–5329. 9188601 10.1128/jvi.71.7.5323-5329.1997 PMC191769

[B213] Pajovic S. Wong E. L. Black A. R. Azizkhan J. C. (1997). Identification of a viral kinase that phosphorylates specific E2Fs and pocket proteins. Mol. Cell. Biol. 17, 6459–6464. 10.1128/MCB.17.11.6459 9343408 PMC232498

[B214] Pardee A. B. (1974). A restriction point for control of normal animal cell proliferation. Proc. Natl. Acad. Sci. U.S.A. 71, 1286–1290. 10.1073/pnas.71.4.1286 4524638 PMC388211

[B215] Park H. U. Jeong J. H. Chung J. H. Brady J. N. (2004). Human T-cell leukemia virus type 1 Tax interacts with Chk1 and attenuates DNA-damage induced G2 arrest mediated by Chk1. Oncogene 23, 4966–4974. 10.1038/sj.onc.1207644 15107832

[B216] Park H. U. Jeong S. J. Jeong J. H. Chung J. H. Brady J. N. (2006). Human T-cell leukemia virus type 1 Tax attenuates gamma-irradiation-induced apoptosis through physical interaction with Chk2. Oncogene 25, 438–447. 10.1038/sj.onc.1209059 16158050

[B217] Peng C. Y. Graves P. R. Thoma R. S. Wu Z. Shaw A. S. Piwnica-Worms H. (1997). Mitotic and G2 checkpoint control: regulation of 14-3-3 protein binding by phosphorylation of Cdc25C on serine-216. Science 277, 1501–1505. 10.1126/science.277.5331.1501 9278512

[B218] Peters J. M. (2006). The anaphase promoting complex/cyclosome: a machine designed to destroy. Nat. Rev. Mol. Cell Biol. 7, 644–656. 10.1038/nrm1988 16896351

[B219] Petri E. T. Errico A. Escobedo L. Hunt T. Basavappa R. (2007). The crystal structure of human cyclin B. Cell Cycle 6, 1342–1349. 10.4161/cc.6.11.4297 17495533

[B220] Pielak R. M. Chou J. J. (2011). Influenza M2 proton channels. Biochim. Biophys. Acta 1808, 522–529. 10.1016/j.bbamem.2010.04.015 20451491 PMC3108042

[B221] Pines J. Rieder C. L. (2001). Re-staging mitosis: a contemporary view of mitotic progression. Nat. Cell Biol. 3:E3–E6. 10.1038/35050676 11146636

[B222] Platt G. Carbone A. Mittnacht S. (2002). p16INK4a loss and sensitivity in KSHV associated primary effusion lymphoma. Oncogene 21, 1823–1831. 10.1038/sj.onc.1205360 11896614

[B223] Poggioli G. J. Dermody T. S. Tyler K. L. (2001). Reovirus-induced sigma1s-dependent G(2)/M phase cell cycle arrest is associated with inhibition of p34(cdc2). J. Virol. 75, 7429–7434. 10.1128/JVI.75.16.7429-7434.2001 11462015 PMC114978

[B224] Poggioli G. J. Keefer C. Connolly J. L. Dermody T. S. Tyler K. L. (2000). Reovirus-induced G(2)/M cell cycle arrest requires sigma 1s and occurs in the absence of apoptosis. J. Virol. 74, 9562–9570. 10.1128/JVI.74.20.9562-9570.2000 11000227 PMC112387

[B225] Poss Z. C. Ebmeier C. C. Taatjes D. J. (2013). The Mediator complex and transcription regulation. Crit. Rev. Biochem. Mol. Biol. 48, 575–608. 10.3109/10409238.2013.840259 24088064 PMC3852498

[B226] Prikhod'ko EA. Miller L. K. (1998). Role of baculovirus IE2 and its RING finger in cell cycle arrest. J. Virol. 72, 684–692. 9420274 10.1128/jvi.72.1.684-692.1998 PMC109423

[B227] Quelle D. E. Ashmun R. A. Shurtleff S. A. Kato J. Y. Bar-Sagi D. Roussel M. F.. (1993). Overexpression of mouse D-type cyclins accelerates G1 phase in rodent fibroblasts. Genes Dev. 7, 1559–1571. 10.1101/gad.7.8.1559 8339933

[B228] Raj K. Ogston P. Beard P. (2001). Virus-mediated killing of cells that lack p53 activity. Nature 412, 914–917. 10.1038/35091082 11528480

[B229] Rane S. G. Dubus P. Mettus R. V. Galbreath E. J. Boden G. Reddy E. P.. (1999). Loss of Cdk4 expression causes insulin-deficient diabetes and Cdk4 activation results in beta-islet cell hyperplasia. Nat. Genet. 22, 44–52. 10.1038/8751 10319860

[B230] Raychaudhuri P. Bagchi S. Devoto S. H. Kraus V. B. Moran E. Nevins J. R. (1991). Domains of the adenovirus E1A protein required for oncogenic activity are also required for dissociation of E2F transcription factor complexes. Genes Dev. 5, 1200–1211. 10.1101/gad.5.7.1200 1829698

[B231] Ren S. Rollins B. J. (2004). Cyclin C/cdk3 promotes Rb-dependent G0 exit. Cell 117, 239–251. 10.1016/S0092-8674(04)00300-9 15084261

[B232] Roshal M. Kim B. Zhu Y. Nghiem P. Planelles V. (2003). Activation of the ATR-mediated DNA damage response by the HIV-1 viral protein R. J. Biol. Chem. 278, 25879–25886. 10.1074/jbc.M303948200 12738771

[B233] Roussel M. F. Davis J. N. Cleveland J. L. Ghysdael J. Hiebert S. W. (1994). Dual control of myc expression through a single DNA binding site targeted by ets family proteins and E2F-1. Oncogene 9, 405–415. 8290253

[B234] Rovnak J. Quackenbush S. L. (2002). Walleye dermal sarcoma virus cyclin interacts with components of the mediator complex and the RNA polymerase II holoenzyme. J. Virol. 76, 8031–8039. 10.1128/JVI.76.16.8031-8039.2002 12134008 PMC155167

[B235] Sakai K. Barnitz R. A. Chaigne-Delalande B. Bidere N. Lenardo M. J. (2011). Human Immunodeficiency Virus Type 1 Vif causes dysfunction of Cdk1 and CyclinB1: implications for cell cycle arrest. Virol. J. 8:219. 10.1186/1743-422X-8-219 21569376 PMC3113979

[B236] Sanchez Y. Wong C. Thoma R. S. Richman R. Wu Z. Piwnica-Worms H.. (1997). Conservation of the Chk1 checkpoint pathway in mammals: linkage of DNA damage to Cdk regulation through Cdc25. Science 277, 1497–1501. 10.1126/science.277.5331.1497 9278511

[B237] Santamaría D. Barriere C. Cerqueira A. Hunt S. Tardy C. Newton K.. (2007). Cdk1 is sufficient to drive the mammalian cell cycle. Nature 448, 811–818. 10.1038/nature06046 17700700

[B238] Sarek G. Jarviluoma A. Ojala P. M. (2006). KSHV viral cyclin inactivates p27KIP1 through Ser10 and Thr187 phosphorylation in proliferating primary effusion lymphomas. Blood 107, 725–732. 10.1182/blood-2005-06-2534 16160006

[B239] Saxena N. Kumar V. (2014). The HBx oncoprotein of hepatitis B virus deregulates the cell cycle by promoting the intracellular accumulation and re-compartmentalization of the cellular deubiquitinase USP37. PLoS ONE 9:e111256. 10.1371/journal.pone.0111256 25347529 PMC4210131

[B240] Schröfelbauer B. Hakata Y. Landau N. R. (2007). HIV-1 Vpr function is mediated by interaction with the damage-specific DNA-binding protein DDB1. Proc. Natl. Acad. Sci. U.S.A. 104, 4130–4135. 10.1073/pnas.0610167104 17360488 PMC1820720

[B241] Schulze-Gahmen U. Jung J. U. Kim S. H. (1999). Crystal structure of a viral cyclin, a positive regulator of cyclin-dependent kinase 6. Structure 7, 245–254. 10.1016/S0969-2126(99)80035-5 10368294

[B242] Seto E. Moosmann A. Groemminger S. Walz N. Grundhoff A. Hammerschmidt W. (2010). Micro RNAs of Epstein-Barr virus promote cell cycle progression and prevent apoptosis of primary human B cells. PLoS Pathog. 6:e1001063. 10.1371/journal.ppat.1001063 20808852 PMC2924374

[B243] Sharon E. Volchek L. Frenkel N. (2014). Human herpesvirus 6 (HHV-6) alters E2F1/Rb pathways and utilizes the E2F1 transcription factor to express viral genes. Proc. Natl. Acad. Sci. U.S.A. 111, 451–456. 10.1073/pnas.1308854110 24335704 PMC3890880

[B244] Shaulsky G. Goldfinger N. Tosky M. S. Levine A. J. Rotter V. (1991). Nuclear-localization is essential for the activity of P53 protein. Oncogene 6, 2055–2065. 1719467

[B245] Sherr C. J. (1993). Mammalian G1 cyclins. Cell 73, 1059–1065. 10.1016/0092-8674(93)90636-5 8513492

[B246] Sherr C. J. Roberts J. M. (1999). CDK inhibitors: positive and negative regulators of G1-phase progression. Genes Dev. 13, 1501–1512. 10.1101/gad.13.12.1501 10385618

[B247] Shieh S. Y. Ahn J. Tamai K. Taya Y. Prives C. (2000). The human homologs of checkpoint kinases Chk1 and Cds1 (Chk2) phosphorylate p53 at multiple DNA damage-inducible sites. Genes Dev. 14, 289–300. 10673501 PMC316358

[B248] Shieh S. Y. Ikeda M. Taya Y. Prives C. (1997). DNA damage-induced phosphorylation of p53 alleviates inhibition by MDM2. Cell 91, 325–334. 10.1016/S0092-8674(00)80416-X 9363941

[B249] Sidle A. Palaty C. Dirks P. Wiggan O. Kiess M. Gill R. M.. (1996). Activity of the retinoblastoma family proteins, pRB, p107, and p130, during cellular proliferation and differentiation. Crit. Rev. Biochem. Mol. Biol. 31, 237–271. 10.3109/10409239609106585 8817077

[B250] Sinclair A. J. Palmero I. Peters G. Farrell P. J. (1994). EBNA-2 and EBNA-LP cooperate to cause G0 to G1 transition during immortalization of resting human B lymphocytes by Epstein-Barr virus. EMBO J. 13, 3321–3328. 10.1002/j.1460-2075.1994.tb06634.x 8045261 PMC395229

[B251] Sinclair J. Baillie J. Bryant L. Caswell R. (2000). Human cytomegalovirus mediates cell cycle progression through G(1) into early S phase in terminally differentiated cells. J. Gen. Virol. 81, 1553–1565. 10.1099/0022-1317-81-6-1553 10811939

[B252] Solomon M. J. Lee T. Kirschner M. W. (1992). Role of phosphorylation in p34cdc2 activation: identification of an activating kinase. Mol. Biol. Cell 3, 13–27. 10.1091/mbc.3.1.13 1532335 PMC275499

[B253] Somasundaram K. El-Deiry W. S. (1997). Inhibition of p53-mediated transactivation and cell cycle arrest by E1A through its p300/CBP-interacting region. Oncogene 14, 1047–1057. 10.1038/sj.onc.1201002 9070653

[B254] Song M. S. Song S. J. Ayad N. G. Chang J. S. Lee J. H. Hong H. K.. (2004). The tumour suppressor RASSF1A regulates mitosis by inhibiting the APC-Cdc20 complex. Nat. Cell Biol. 6, 129–137. 10.1038/ncb1091 14743218

[B255] Souza B. S. Sampaio G. L. Pereira C. S. Campos G. S. Sardi S. I. Freitas L. A.. (2016). Zika virus infection induces mitosis abnormalities and apoptotic cell death of human neural progenitor cells. Sci. Rep. 6:39775. 10.1038/srep39775 28008958 PMC5180086

[B256] Speir E. Modali R. Huang E. S. Leon M. B. Shawl F. Finkel T.. (1994). Potential role of human cytomegalovirus and p53 interaction in coronary restenosis. Science 265, 391–394. 10.1126/science.8023160 8023160

[B257] Stambolic V. MacPherson D. Sas D. Lin Y. Snow B. Jang Y.. (2001). Regulation of PTEN transcription by p53. Mol. Cell 8, 317–325. 10.1016/S1097-2765(01)00323-9 11545734

[B258] Straight A. F. Field C. M. (2000). Microtubules, membranes and cytokinesis. Curr. Biol. 10, R760–R770. 10.1016/S0960-9822(00)00746-6 11069103

[B259] Strausfeld U. Labbe J. C. Fesquet D. Cavadore J. C. Picard A. Sadhu K.. (1991). Dephosphorylation and activation of a p34cdc2/cyclin B complex *in vitro* by human CDC25 protein. Nature 351, 242–245. 10.1038/351242a0 1828290

[B260] Stubdal H. Zalvide J. DeCaprio J. A. (1996). Simian virus 40 large T antigen alters the phosphorylation state of the RB-related proteins p130 and p107. J. Virol. 70, 2781–2788. 8627752 10.1128/jvi.70.5.2781-2788.1996 PMC190135

[B261] Sudakin V. Ganoth D. Dahan A. Heller H. Hershko J. Luca F. C.. (1995). The cyclosome, a large complex containing cyclin-selective ubiquitin ligase activity, targets cyclins for destruction at the end of mitosis. Mol. Biol. Cell 6, 185–197. 10.1091/mbc.6.2.185 7787245 PMC275828

[B262] Sullivan C. S. Cantalupo P. Pipas J. M. (2000). The molecular chaperone activity of simian virus 40 large T antigen is required to disrupt Rb-E2F family complexes by an ATP-dependent mechanism. Mol. Cell. Biol. 20, 6233–6243. 10.1128/MCB.20.17.6233-6243.2000 10938100 PMC86098

[B263] Sun Y. Chen A. Y. Cheng F. Guan W. Johnson F. B. Qiu J. (2009). Molecular characterization of infectious clones of the minute virus of canines reveals unique features of bocaviruses. J. Virol. 83, 3956–3967. 10.1128/JVI.02569-08 19211770 PMC2663281

[B264] Suzuki A. Hayashida M. Ito T. Kawano H. Nakano T. Miura M.. (2000). Survivin initiates cell cycle entry by the competitive interaction with Cdk4/p16(INK4a) and Cdk2/cyclin E complex activation. Oncogene 19, 3225–3234. 10.1038/sj.onc.1203665 10918579

[B265] Suzuki T. Kitao S. Matsushime H. Yoshida M. (1996). HTLV-1 Tax protein interacts with cyclin-dependent kinase inhibitor p16INK4A and counteracts its inhibitory activity towards CDK4. EMBO J. 15, 1607–1614. 10.1002/j.1460-2075.1996.tb00505.x 8612584 PMC450070

[B266] Suzuki T. Narita T. Uchida-Toita M. Yoshida M. (1999). Down-regulation of the INK4 family of cyclin-dependent kinase inhibitors by tax protein of HTLV-1 through two distinct mechanisms. Virology 259, 384–391. 10.1006/viro.1999.9760 10388662

[B267] Svendsen J. M. Smogorzewska A. Sowa M. E. O'Connell B. C. Gygi S. P. Elledge S. J.. (2009). Mammalian BTBD12/SLX4 assembles a Holliday junction resolvase and is required for DNA repair. Cell 138, 63–77. 10.1016/j.cell.2009.06.030 19596235 PMC2720686

[B268] Swanton C. Mann D. J. Fleckenstein B. Neipel F. Peters G. Jones N. (1997). Herpes viral cyclin/Cdk6 complexes evade inhibition by CDK inhibitor proteins. Nature 390, 184–187. 10.1038/36606 9367157

[B269] Szekely L. Selivanova G. Magnusson K. P. Klein G. Wiman K. G. (1993). EBNA-5, an Epstein-Barr virus-encoded nuclear antigen, binds to the retinoblastoma and p53 proteins. Proc. Natl. Acad. Sci. U.S.A. 90, 5455–5459. 10.1073/pnas.90.12.5455 8390666 PMC46739

[B270] Tachiwana H. Shimura M. Nakai-Murakami C. Tokunaga K. Takizawa Y. Sata T.. (2006). HIV-1 Vpr induces DNA double-strand breaks. Cancer Res. 66, 627–631. 10.1158/0008-5472.CAN-05-3144 16423988

[B271] Tan T. H. Wallis J. Levine A. J. (1986). Identification of the p53 protein domain involved in formation of the simian virus-40 large T-antigen p53 protein complex. J. Virol. 59, 574–583. 3016321 10.1128/jvi.59.3.574-583.1986 PMC253211

[B272] Tang H. Hammack C. Ogden S. C. Wen Z. Qian X. Li Y.. (2016). Zika virus infects human cortical neural progenitors and attenuates their growth. Cell Stem Cell 18, 587–590. 10.1016/j.stem.2016.02.016 26952870 PMC5299540

[B273] Tang Z. Li B. Bharadwaj R. Zhu H. Ozkan E. Hakala K.. (2001). APC2 Cullin protein and APC11 RING protein comprise the minimal ubiquitin ligase module of the anaphase-promoting complex. Mol. Biol. Cell 12, 3839–3851. 10.1091/mbc.12.12.3839 11739784 PMC60759

[B274] Tassan J. P. Jaquenoud M. Leopold P. Schultz S. J. Nigg E. A. (1995). Identification of human cyclin-dependent kinase 8, a putative protein kinase partner for cyclin C. Proc. Natl. Acad. Sci. U.S.A. 92, 8871–8875. 10.1073/pnas.92.19.8871 7568034 PMC41069

[B275] Teodoro J. G. Heilman D. W. Parker A. E. Green M. R. (2004). The viral protein Apoptin associates with the anaphase-promoting complex to induce G2/M arrest and apoptosis in the absence of p53. Genes Dev. 18, 1952–1957. 10.1101/gad.1198404 15314021 PMC514174

[B276] Thompson D. L. Kalderon D. Smith A. E. Tevethia M. J. (1990). Dissociation of Rb-binding and anchorage-independent growth from immortalization and tumorigenicity using SV40 mutants producing N-terminally truncated large T antigens. Virology 178, 15–34. 10.1016/0042-6822(90)90375-2 2167547

[B277] Thompson J. Doniger J. Rosenthal L. J. (1994). A 79 amino acid oncogene is responsible for human cytomegalovirus mtrII induced malignant transformation. Arch. Virol. 136, 161–172. 10.1007/BF01538825 8002783

[B278] Thornton B. R. Toczyski D. P. (2003). Securin and B-cyclin/CDK are the only essential targets of the APC. Nat. Cell Biol. 5, 1090–1094. 10.1038/ncb1066 14634663

[B279] Tsai H. L. Kou G. H. Chen S. C. Wu C. W. Lin Y. S. (1996). Human cytomegalovirus immediate-early protein IE2 tethers a transcriptional repression domain to p53. J. Biol. Chem. 271, 3534–3540. 10.1074/jbc.271.7.3534 8631958

[B280] Tsutsui T. Fukasawa R. Tanaka A. Hirose Y. Ohkuma Y. (2011). Identification of target genes for the CDK subunits of the Mediator complex. Genes Cells 16, 1208–1218. 10.1111/j.1365-2443.2011.01565.x 22117896

[B281] Tsutsui T. Hesabi B. Moons D. S. Pandolfi P. P. Hansel K. S. Koff A.. (1999). Targeted disruption of CDK4 delays cell cycle entry with enhanced p27(Kip1) activity. Mol. Cell. Biol. 19, 7011–7019. 10.1128/MCB.19.10.7011 10490638 PMC84696

[B282] Tsutsui T. Umemura H. Tanaka A. Mizuki F. Hirose Y. Ohkuma Y. (2008). Human mediator kinase subunit CDK11 plays a negative role in viral activator VP16-dependent transcriptional regulation. Genes Cells 13, 817–826. 10.1111/j.1365-2443.2008.01208.x 18651850

[B283] Uhlmann T. Boeing S. Lehmbacher M. Meisterernst M. (2007). The VP16 activation domain establishes an active mediator lacking CDK8 *in vivo*. J. Biol. Chem. 282, 2163–2173. 10.1074/jbc.M608451200 17135252

[B284] van Dyk L. F. Hess J. L. Katz J. D. Jacoby M. Speck S. H. Virgin H. I. (1999). The murine gammaherpesvirus 68 v-cyclin gene is an oncogene that promotes cell cycle progression in primary lymphocytes. J. Virol. 73, 5110–5122. 10233974 10.1128/jvi.73.6.5110-5122.1999 PMC112556

[B285] Virgin H. W. T. Latreille P. Wamsley P. Hallsworth K. Weck K. E. Dal Canto A. J.. (1997). Complete sequence and genomic analysis of murine gammaherpesvirus 68. J. Virol. 71, 5894–5904. 9223479 10.1128/jvi.71.8.5894-5904.1997 PMC191845

[B286] Vlach J. Hennecke S. Amati B. (1997). Phosphorylation-dependent degradation of the cyclin-dependent kinase inhibitor p27. EMBO J. 16, 5334–5344. 10.1093/emboj/16.17.5334 9311993 PMC1170165

[B287] von Mikecz A. (2006). The nuclear ubiquitin-proteasome system. J. Cell Sci. 119, 1977–1984. 10.1242/jcs.03008 16687735

[B288] Wan Z. Zhi N. Wong S. Keyvanfar K. Liu D. Raghavachari N.. (2010). Human parvovirus B19 causes cell cycle arrest of human erythroid progenitors via deregulation of the E2F family of transcription factors. J. Clin. Invest. 120, 3530–3544. 10.1172/JCI41805 20890043 PMC2947219

[B289] Wang F. Yoshida I. Takamatsu M. Ishido S. Fujita T. Oka K.. (2000). Complex formation between hepatitis C virus core protein and p21Waf1/Cip1/Sdi1. Biochem. Biophys. Res. Commun. 273, 479–484. 10.1006/bbrc.2000.2970 10873631

[B290] Wang F. Zhou H. Xia X. Sun Q. Wang Y. Cheng B. (2010). Activated Notch signaling is required for hepatitis B virus X protein to promote proliferation and survival of human hepatic cells. Cancer Lett. 298, 64–73. 10.1016/j.canlet.2010.06.003 20638778

[B291] Wang J. Belcher J. D. Marker P. H. Wilcken D. E. Vercellotti G. M. Wang X. L. (2001). Cytomegalovirus inhibits p53 nuclear localization signal function. J. Mol. Med. 78, 642–647. 10.1007/s001090000157 11269511

[B292] Wang X. Deng X. Yan W. Zhu Z. Shen Y. Qiu Y.. (2012). Stabilization of p53 in influenza A virus-infected cells is associated with compromised MDM2-mediated ubiquitination of p53. J. Biol. Chem. 287, 18366–18375. 10.1074/jbc.M111.335422 22474335 PMC3365762

[B293] Wehman A. M. Staub W. Baier H. (2007). The anaphase-promoting complex is required in both dividing and quiescent cells during zebrafish development. Dev. Biol. 303, 144–156. 10.1016/j.ydbio.2006.10.043 17141209

[B294] Wei W. Ayad N. G. Wan Y. Zhang G. J. Kirschner M. W. Kaelin W. G. (2004). Degradation of the SCF component Skp2 in cell-cycle phase G1 by the anaphase-promoting complex. Nature 428, 194–198. 10.1038/nature02381 15014503

[B295] Weinberg R. A. (1991). Tumor suppressor genes. Science 254, 1138–1146. 10.1126/science.1659741 1659741

[B296] Weinberg R. A. (1995). The retinoblastoma protein and cell cycle control. Cell 81, 323–330. 10.1016/0092-8674(95)90385-2 7736585

[B297] Weinberg R. A. (1997). The cat and mouse games that genes, viruses, and cells play. Cell 88, 573–575. 10.1016/S0092-8674(00)81897-8 9054495

[B298] Werness B. A. Levine A. J. Howley P. M. (1990). Association of human papillomavirus types 16 and 18 E6 proteins with p53. Science 248, 76–79. 10.1126/science.2157286 2157286

[B299] Wittschieben B. O. Wood R. D. (2003). DDB complexities. DNA Repair (Amst). 2, 1065–1069. 10.1016/S1568-7864(03)00113-7 12967661

[B300] Wu E. W. Clemens K. E. Heck D. V. Munger K. (1993). The human papillomavirus E7 oncoprotein and the cellular transcription factor E2F bind to separate sites on the retinoblastoma tumor suppressor protein. J. Virol. 67, 2402–2407. 8445736 10.1128/jvi.67.4.2402-2407.1993 PMC240412

[B301] Xiao Z. Chen Z. Gunasekera A. H. Sowin T. J. Rosenberg S. H. Fesik S.. (2003). Chk1 mediates S and G2 arrests through Cdc25A degradation in response to DNA-damaging agents. J. Biol. Chem. 278, 21767–21773. 10.1074/jbc.M300229200 12676925

[B302] Xu J. Yun X. Jiang J. Wei Y. Wu Y. Zhang W.. (2010). Hepatitis B virus X protein blunts senescence-like growth arrest of human hepatocellular carcinoma by reducing Notch1 cleavage. Hepatology 52, 142–154. 10.1002/hep.23613 20578140

[B303] Yanagida M. (2000). Cell cycle mechanisms of sister chromatid separation; Roles of Cut1/separin and Cut2/securin. Genes Cells 5, 1–8. 10.1046/j.1365-2443.2000.00306.x 10651900

[B304] Yang M. Hay J. Ruyechan W. T. (2008). Varicella-zoster virus IE62 protein utilizes the human mediator complex in promoter activation. J. Virol. 82, 12154–12163. 10.1128/JVI.01693-08 18842726 PMC2593350

[B305] Yarmishyn A. Child E. S. Elphick L. M. Mann D. J. (2008). Differential regulation of the cyclin-dependent kinase inhibitors p21(Cip1) and p27(Kip1) by phosphorylation directed by the cyclin encoded by Murine Herpesvirus 68. Exp. Cell Res. 314, 204–212. 10.1016/j.yexcr.2007.09.016 17997402

[B306] Ye X. Zhu C. H. Harper J. W. (2001). A premature-termination mutation in the Mus musculus cyclin-dependent kinase 3 gene. Proc. Natl. Acad. Sci. U.S.A. 98, 1682–1686. 10.1073/pnas.98.4.1682 11172011 PMC29317

[B307] Yip K. W. Shi W. Pintilie M. Martin J. D. Mocanu J. D. Wong D.. (2006). Prognostic significance of the Epstein-Barr virus, p53, Bcl-2, and survivin in nasopharyngeal cancer. Clin. Cancer Res. 12, 5726–5732. 10.1158/1078-0432.CCR-06-0571 17020977

[B308] Yu J. Zhang L. Ren P. Zhong T. Li Z. Wang Z.. (2015). Enterovirus 71 mediates cell cycle arrest in S phase through non-structural protein 3D. Cell Cycle 14, 425–436. 10.4161/15384101.2014.980631 25659038 PMC4353240

[B309] Zacny V. L. Wilson J. Pagano J. S. (1998). The Epstein-Barr virus immediate-early gene product, BRLF1, interacts with the retinoblastoma protein during the viral lytic cycle. J. Virol. 72, 8043–8051. 9733844 10.1128/jvi.72.10.8043-8051.1998 PMC110141

[B310] Zalvide J. Stubdal H. DeCaprio J. A. (1998). The J domain of simian virus 40 large T antigen is required to functionally inactivate RB family proteins. Mol. Cell. Biol. 18, 1408–1415. 10.1128/MCB.18.3.1408 9488456 PMC108854

[B311] Zerfass-Thome K. Zwerschke W. Mannhardt B. Tindle R. Botz J. W. Jansen-Durr P. (1996). Inactivation of the cdk inhibitor p27KIP1 by the human papillomavirus type 16 E7 oncoprotein. Oncogene 13, 2323–2330. 8957073

[B312] Zetterberg A. Larsson O. Wiman K. G. (1995). What is the restriction point. Curr. Opin. Cell Biol. 7, 835–842. 10.1016/0955-0674(95)80067-0 8608014

[B313] Zhang Y. Lu H. (2009). Signaling to p53: ribosomal proteins find their way. Cancer Cell 16, 369–377. 10.1016/j.ccr.2009.09.024 19878869 PMC4369769

[B314] Zhao F. Hou N. B. Yang X. L. He X. Liu Y. Zhang Y. H.. (2008). Ataxia telangiectasia-mutated-Rad3-related DNA damage checkpoint signaling pathway triggered by hepatitis B virus infection. World J. Gastroenterol. 14, 6163–6170. 10.3748/wjg.14.6163 18985806 PMC2761577

[B315] Zhao H. Piwnica-Worms H. (2001). ATR-mediated checkpoint pathways regulate phosphorylation and activation of human Chk1. Mol. Cell. Biol. 21, 4129–4139. 10.1128/MCB.21.13.4129-4139.2001 11390642 PMC87074

[B316] Zhao H. Watkins J. L. Piwnica-Worms H. (2002). Disruption of the checkpoint kinase 1/cell division cycle 25A pathway abrogates ionizing radiation-induced S and G2 checkpoints. Proc. Natl. Acad. Sci. U.S.A. 99, 14795–14800. 10.1073/pnas.182557299 12399544 PMC137498

[B317] Zhi H. Zahoor M. A. Shudofsky A. M. Giam C. Z. (2015). KSHV vCyclin counters the senescence/G1 arrest response triggered by NF-kappaB hyperactivation. Oncogene 34, 496–505. 10.1038/onc.2013.567 24469036 PMC4112183

[B318] Zhou Y. Ching Y. P. Ng R. W. M. Jin D. Y. (2003). Differential expression, localization and activity of two alternatively spliced isoforms of human APC regulator CDH1. Biochem. J. 374, 349–358. 10.1042/bj20030600 12797865 PMC1223613

[B319] Zimmerman E. S. Sherman M. P. Blackett J. L. Neidleman J. A. Kreis C. Mundt P.. (2006). Human immunodeficiency virus type 1 Vpr induces DNA replication stress *in vitro* and *in vivo*. J. Virol. 80, 10407–10418. 10.1128/JVI.01212-06 16956949 PMC1641771

[B320] Zou H. McGarry T. J. Bernal T. Kirschner M. W. (1999). Identification of a vertebrate sister-chromatid separation inhibitor involved in transformation and tumorigenesis. Science 285, 418–422. 10.1126/science.285.5426.418 10411507

[B321] Zou L. Elledge S. J. (2003). Sensing DNA damage through ATRIP recognition of RPA-ssDNA complexes. Science 300, 1542–1548. 10.1126/science.1083430 12791985

[B322] zur Hausen H. (2001). Oncogenic DNA viruses. Oncogene 20, 7820–7823. 10.1038/sj.onc.1204958 11753664

